# Quantifying and adjusting for selection biases in the Norwegian Mother, Father and Child Cohort Study using population-wide individual-level registry information

**DOI:** 10.1093/ije/dyag122

**Published:** 2026-07-25

**Authors:** Christopher Rayner, Laurie J Hannigan, Isabella Badini, Perline A Demange, Sverre Berg Ofstad, Eivind Ystrom, Tom A McAdams

**Affiliations:** Social, Genetic and Developmental Psychiatry Centre, Institute of Psychiatry, Psychology and Neuroscience, King’s College London, London, United Kingdom; Research Department, Lovisenberg Diaconal Hospital, Oslo, Norway; PsychGen Centre for Genetic Epidemiology and Mental Health, Norwegian Institute of Public Health, Oslo, Norway; Division of Psychiatry, University College London, London, United Kingdom; PROMENTA Research Center, Department of Psychology, University of Oslo, Oslo, Norway; PROMENTA Research Center, Department of Psychology, University of Oslo, Oslo, Norway; PROMENTA Research Center, Department of Psychology, University of Oslo, Oslo, Norway; Centre for Research on Equality in Education, University of Oslo, Oslo, Norway; Department for Child Health and Development, Norwegian Institute of Public Health, Oslo, Norway; Social, Genetic and Developmental Psychiatry Centre, Institute of Psychiatry, Psychology and Neuroscience, King’s College London, London, United Kingdom

**Keywords:** MoBa, participation, attrition, inverse probability weighting, observational studies, target population

## Abstract

**Background:**

Selective participation in research studies hampers researchers’ ability to draw valid and generalizable inferences from analyses. Quantifying and adjusting for selective participation are desirable but can be challenging given the paucity of data for non-participants.

**Methods:**

We used individual-level information from population registers to predict baseline and continued participation in the Norwegian Mother, Father and Child Cohort Study (MoBa). Inverse probability weights were computed from logistic regression models with elastic-net regularization. We predicted selective participation and attrition in 296 987 mothers, of whom 29% returned the first MoBa questionnaire and 12.5% returned a follow-up questionnaire 8 years later. To quantify bias, we computed sample characteristics and exposure–outcome associations in the target population and stratified samples. To compare approaches for adjusting for bias, we computed weighted sample estimates by using three sets of weights.

**Results:**

Unweighted sample estimates were systematically different from target-population values, indicating bias due to selective participation. Baseline participation weights substantially reduced the impact of selection bias on mean values by 93% and associations by 75%. Attrition weights reduced attrition bias on mean values by 78% and associations by 50% but were less effective for reducing participation bias (22% and 14% respectively).

**Conclusion:**

In this sample, characteristics and effect estimates are substantially different from target-population values. Participation weights were relatively effective at reducing bias due to selective participation but attrition weights were not. Estimates from studies that use attrition weights may still contain non-negligible selection bias—particularly if the baseline sample is not representative of the target population. Future studies should prioritize opportunities for deriving sampling weights for participation as well as attrition.

Key Messages

- We investigated whether the Norwegian Mother, Father and Child Cohort Study (MoBa) sample characteristics and effect estimates were representative of the target population due to sample selection and used Norwegian population registry data to estimate bias and compute sampling weights.
- Participation and attrition biases were pervasive but baseline participation weights reduced the impact of participation bias on mean values (93%) and associations (75%); however, attrition weights were less effective at reducing attrition bias (78% and 50%) and baseline participation bias (22% and 14%).
- We recommend that researchers using MoBa should also use Norwegian population registry data to compute the sampling weights for their analytical sample.

## Introduction

Prospective cohort studies are major contributors to epidemiological research literature [1, 2]. However, healthy individuals from advantaged socio-economic circumstances are more likely to participate in research studies [3] and provide more complete and accurate information, over a longer period of time [4–6]. Therefore, individuals with poor health and greater exposure to socio-economic disadvantages are under-represented in analytic samples when compared with the ‘target population’ (i.e. the complete group of individuals to whom researchers want to generalize their conclusions).

When analysis samples are not representative of the target population, bias ‘can’ be introduced into sample estimates, which may impact the generalizability of the findings. However, sample representativeness, which is achieved when the distribution of characteristics within the analytic sample mirrors that of the target population, differs from estimate representativeness, which pertains to whether the effect estimate derived from the sample is an unbiased measure of the true effect in that target population [7]. Thus, generalizability is only limited when sample non-representativeness gives rise to estimate non-representativeness (i.e. selection bias).

**Figure 1:**
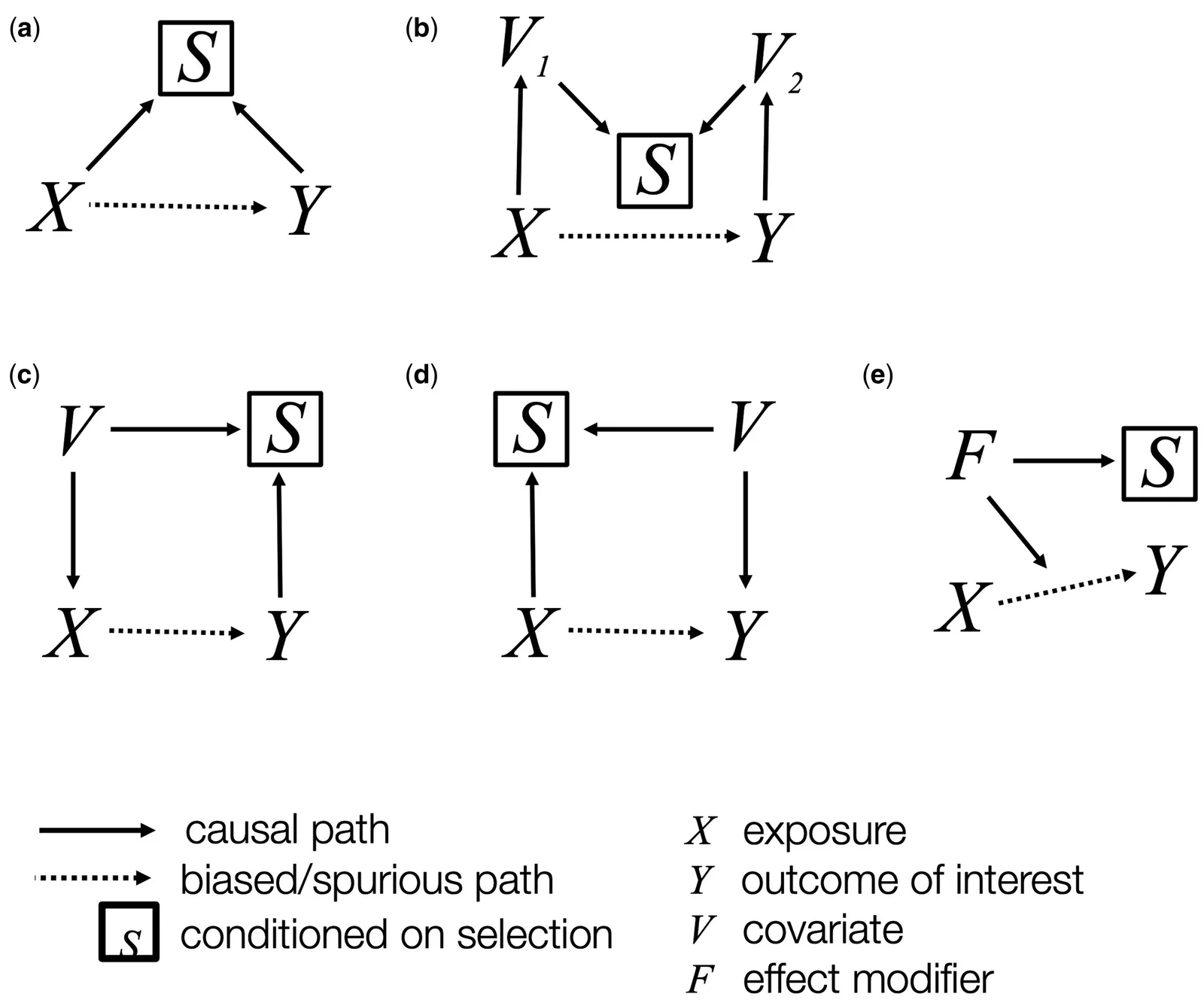
Directed acyclic graphs depicting the way in which conditioning on sample selection can introduce bias into exposure–outcome relationships. Type 1 selection bias (collider restriction bias) occurs because variables that influence selection (S) are conditioned upon. This can occur when both the exposure (X) and the outcome (Y) cause sample selection (S) (a) directly or (b, c, d) indirectly via observed or unobserved covariates (V). This biases the association between the exposure (X) and the outcome (Y), and the bias is proportional to the strength of the association between X and S, and Y and S. (e) Type 2 selection bias, also called generalizability bias, occurs if the effect of X depends on another variable (i.e. an effect modifier, F) that is also related to the selection and bias is introduced when the X, Y, and F distributions differ between participants and non-participants.

Two types of selection bias have been delineated [8, 9] (see Fig. 1). Type 1 is caused by conditioning on a collider variable (‘collider restriction bias’). If individual-level characteristics associated with sample selection are also associated with the exposure or the outcome of interest, then this can lead to biased associations between the exposure and the outcome [10]. Type 2 occurs when the effect of the exposure on the outcome depends on another variable (i.e. an effect modifier) that is also related to selection (‘generalizability bias’). In this scenario, estimates will depend on the distribution of effect modifiers in the selected sample [11]. However, generalizability can be achieved if differences between the analytic sample and the target population can be accounted for by using analytical methods [7, 11, 12]. Crucially, selection bias can arise at multiple stages: first, during initial recruitment from the target population to the study sample (participation bias) and, second, during the transition from the baseline study sample to the final analytic sample (attrition bias). Where both distinct selection processes are operating, they require quantification and adjustment.

Due to lack of access to individual-level data from the target population or sampling weights, studies often present unweighted analyses and rarely assess the potential impact of participation bias. Some studies have used summary data from the target population to construct sampling weights and report reductions in participation bias [9, 13–16]. However, comparisons to the target population are limited to a small selection of variables and representativeness on these specific traits does not guarantee that the sample is representative across all other variables of interest. Some studies use baseline study data to correct for attrition over time, which may correct for attrition bias, but it is rarely possible to compare with target-population values to assess whether estimates are representative. The linkage of cohort samples and population-wide registry data offers new opportunities to explore the impact of selection bias, including the different roles of baseline and continued participation.

Here, we use individual-level information from Norwegian population registers to investigate the socio-demographic profile of the Norwegian Mother, Father and Child Cohort Study (MoBa). We quantify bias in means and prevalence (sample representativeness) and exposure–outcome associations (estimate representativeness), comparing the target population and participants at baseline (15th week of pregnancy) and at follow-up (8 years later). We then use population-wide individual-level data to compute statistical models predicting participation in MoBa from the target population. Inverse probability of participation weights are computed from these models and used to improve the representativeness of sample estimates. In this paper, we aim both to describe the extent to which MoBa is affected by selection bias from baseline and continued participation, and to demonstrate correction for this bias by using sampling weights derived from population-wide individual-level data.

## Methods

### Samples

#### MoBa participants

**Figure 2:**
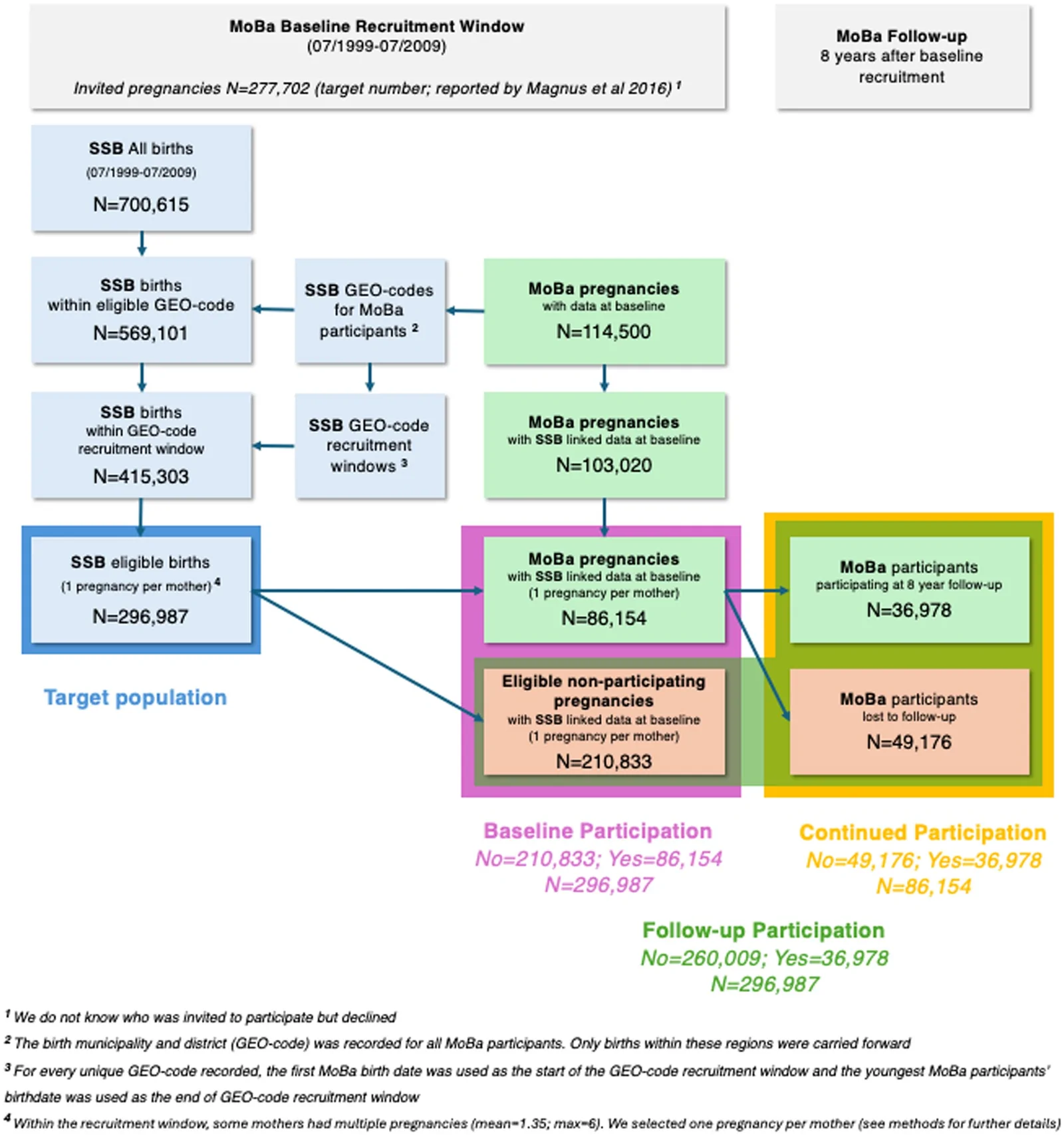
Eligibility inclusion criteria, phenotype definitions, and sample sizes for the target population, and baseline, follow-up, and continued-participation samples. The chart shows three main flows. The target population (dark-blue box) starts with all births in the population register within the full MoBa recruitment window (‘SSB [Statistics Norway] All births’; *N* = 700 615) and is filtered by geographical location of birth (‘eligible geo-code’; *N* = 569 101) and the geographically linked recruitment window (*N* = 415 303) to arrive at the target population of eligible births filtering to one pregnancy per mother (*N* = 296 987). Baseline participation (dark-pink box) includes ‘MoBa pregnancies (one pregnancy per mother)’ with data at baseline (participants; *N* = 86 154) and non-participating mothers from the target population (non-participants; *N* = 210 833). For follow-up participation (dark-green box), the baseline sample (*N* = 86 154) is shown to split into ‘MoBa participants participating at 8-year follow-up’ (*N* = 36 978) and ‘Eligible non-participating participants lost to follow-up’ (*N* = 49 176), which are combined with the non-participants from baseline into *N* = 260 009 non-participants at follow-up. For continued participation (orange box), the baseline sample (*N* = 86 154) is shown to split into ‘MoBa participants participating at 8-year follow-up’ (*N* = 36 978) and ‘Eligible non-participating participants lost to follow-up’ (*N* = 49 176).

The MoBa Study is a prospective population-based pregnancy cohort study conducted by the Norwegian Institute of Public Health [17]. During the recruitment period (1999–2009), invitations were sent to women in 277 702 pregnancies, with a baseline participation rate of 41% (*N* = 114 500) [17]. After linkage with Norwegian population registry data from Statistisk Sentralbyrå (SSB; Statistics Norway), we identified 103 020 parent–offspring trios with MoBa data at baseline—<114 500 known to be in MoBa. The missingness was attributable either to missing IDs for linkage (*N* = 1499) or because they did not complete the baseline questionnaire (*N* = 10 350, included as non-participants; Fig. 2 and Supplementary file 1).

#### Target population

The target population was drawn from SSB data—a national register containing information about all pregnancies in Norway. A subset of pregnant women who were likely to have been invited to participate was determined by using geographically linked recruitment windows. Mothers were restricted to one pregnancy in the data and earlier pregnancies were prioritized (*N* = 296 987; Fig. 2).

### Variables

#### Participation in MoBa

Baseline participation in MoBa (15th week of pregnancy) was defined within the target population by the return of the baseline MoBa questionnaire (0/1). Follow-up participation in MoBa was defined from mothers returning the follow-up questionnaire 8 years later (0/1). We analysed: baseline participation (population vs. baseline participants), follow-up participation (population vs. follow-up participants), and continued participation (baseline vs. follow-up participants; see Fig. 2).

#### Predictors of participation in MoBa used to compute sampling weights

Socio-demographic and geographical data for mothers and fathers were obtained from SSB datasets (Supplementary file 2). Demographic variables included age, civil status, birth place, number of Norwegian-born parents, municipality of current residence, municipality of current workplace, and urbanicity of district of current residence. Social predictors included multiple indicators of income (Norwegian Krone; adjusted for inflation; log-transformed), education (see https://www.ssb.no/en/klass/klassifikasjoner/36), and occupation (see https://www.ssb.no/en/klass/klassifikasjoner/7). For each pregnancy, variables were extracted for the year of delivery and the preceding and succeeding years, and the average value across these years was carried forward. Continuous variables were winsorized and standardized (M = 0, SD = 1). All categorical variables were dummy coded by using the most common category as the reference category. To optimize computation and avoid overfitting, dummy-coded variables with <2% of the prediction sample in either level were dropped. In total, 221 variables were included in the prediction models. The levels of missing data are displayed in Supplementary file 2. Where we had incomplete data on predictors of participation, we performed a single imputation of missing values through random forest regression and classification models using ‘mlim’ [18]. Single imputation allowed us to derive a single sampling weight that could be applied to demonstrate the impact of selection bias in this sample and the efficacy of reweighting analyses. Further details of our approach are provided in Supplementary file 2.

#### Phenotypes for bias quantification and adjustment

To quantify sample and estimate representativeness, we compared means, variances, and outcome–exposure associations between the target population, the baseline sample, the follow-up sample, and the continued-participation sample. To assess sample representativeness, we selected a range of socio-demographic variables (age, birth place, immigration status, education, income, employment, hours worked, and hometown urbanicity), as well as other variables of interest. These included children’s standardized national test scores (averaged across English and mathematics at ages 5, 8, and 9 years), extracted from Norway’s National Education Database. We also looked at mental health and neurodevelopmental outcomes, as these conditions are more prevalent in this age group (compared with common diseases of ageing). We used ICD10 and ICPC2 codes recorded from the Norwegian Control and Payment of Health Reimbursements Database (KUHR; 2006–23; Supplementary file 2) to compute ‘probable’ diagnosis of (i) an internalizing disorder (a phobia, anxiety, or depressive disorder) and (ii) attention-deficit hyperactivity disorder (ADHD). We describe these diagnoses as ‘probable’ because the diagnostic codes can be entered from a range of healthcare visits and are not necessarily derived by using a clinical instrument. Unweighted and weighted means were estimated for this variable set.

For exposure–outcome associations, we selected two outcomes: mothers’ household income and children’s national test scores. For mothers’ household income, we tested for univariable associations with mothers’ age, birthplace (Norway: 0/1), civil status (married: 0/1), number of years of education, average weekly hours worked, living in an urban area (0/1), having an internalizing diagnosis (0/1), and having an ADHD diagnosis (0/1). For children’s national test scores, we estimated the univariable associations of children’s sex (male/female), number of Norwegian parents (0,1,2), having a probable internalizing diagnosis (0/1), having a probable ADHD diagnosis (0/1), as well as their mothers’ household income (M = 0, SD = 1), number of years of education (M = 0, SD = 1), civil status (married: 0/1), having a probable internalizing diagnosis (0/1), and having a probable ADHD diagnosis (0/1).

### Statistical modelling

#### Identifying predictors of participation

Three prediction models were estimated for three sets of comparisons. Following previous studies [14, 16] and given the large number of variables available in the population registers, we used elastic-net regularized logistic regression to select the optimal combination of predictors. We computed the models by using the sparse logistic regression function from the ‘bigstatsr’ package [19], which uses cross-model selection and averaging to perform a procedure similar to cross-validation to choose hyper-parameters of the elastic-net regularization (Supplementary file).

#### Deriving inverse probability of participation weights

Using the predictor set identified in the penalized regression models, we computed the predicted probabilities of baseline participation (population vs. baseline participants; *P*_BL_), follow-up participation (population vs. follow-up participants; *P*_FU_), and continued participation (baseline vs. follow-up participants; i.e. attrition; *P*_ATT_). These predicted probabilities were used to derive sets of inverse probability weights (IPW) for MoBa participants (*IPW*_BL_, *IPW*_FU_, *IPW*_ATT_). Predicted probabilities (*P*) were computed from model outputs and the IPW was computed as the reciprocal of *P: IPW* = $\frac{1}{P}$.

#### Unweighted and weighted estimates

We computed means for selected variables, comparing: target-population reference values; unweighted sample estimates; and weighted sample estimates. For unweighted analyses, we used R stats [20] and, for weighted analyses (weighted means and weighted least-squares regression), we used the survey package [21].

#### Quantifying biases and comparing adjustment strategies in MoBa

Pairwise differences were quantified by using Cohen’s d [22] (means), Cohen’s h (proportions), or the difference between standardized univariable regression coefficients (associations). We tested for mean and association differences via two-tailed two-sample Z-tests using Welch–Satterthwaite’s degrees of freedom (df). Pairwise proportions were tested by using the Wald statistic and *χ*^2^ tests with 1 df. Principal component analysis on the dataset with analysis variables indicated that 17 principal components were sufficient to explain 99.5% of the variance in the data and thus our Bonferroni adjusted *P* value was .05/17 = .00294. For each set of analyses, we computed the average standardized difference as a summary indicator of the bias.

## Results

### Sample characteristics

**Figure 3:**
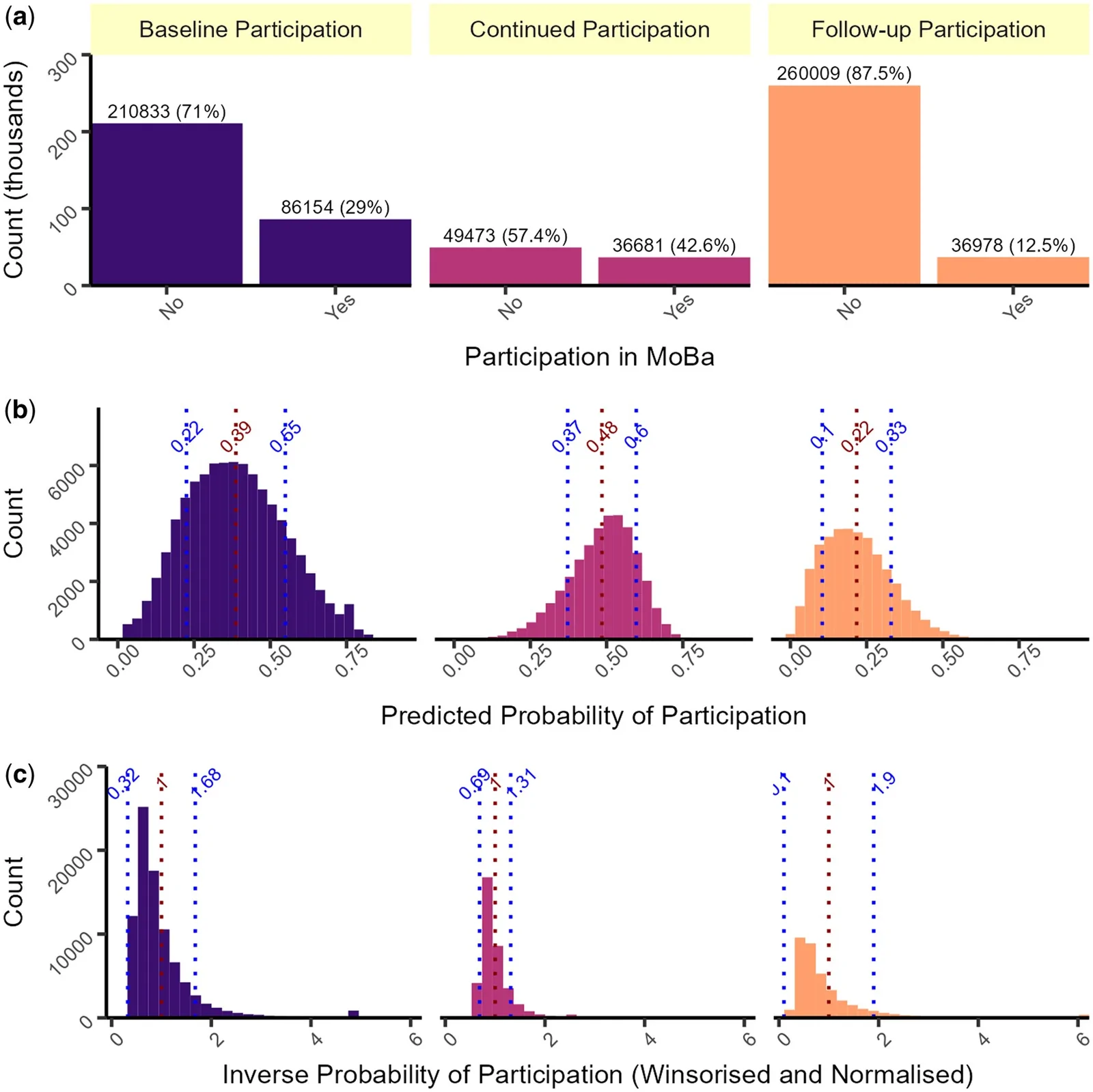
(a) Counts and percentages for participation at the MoBa baseline and follow-up participation (nested in the eligible population) and continued participation (follow-up nested in baseline participants). (b) Predicted probabilities of participation for each of the outcomes estimated from logistic regression models with elastic-net regularization. (C) Inverse probability of participation weights computed from predicted probabilities. Mean values labelled in red and 1 SD intervals are labelled in blue.

The participation outcome distributions are displayed in Fig. 3. In the target population (*N* = 296 987), the baseline participation outcome included the 210 833 non-participating mothers and the 86 154 MoBa mothers who returned the baseline MoBa questionnaire. Again, in the target population (*N* = 296 987), the follow-up participation outcome included the 260 009 non-participating and attritioned mothers, and the 36 978 MoBa mothers who returned the 8-year MoBa follow-up questionnaire. Finally, the continued-participation outcome was derived from 86 154 MoBa mothers who returned the baseline MoBa questionnaire, where 49 176 had been attritioned and 36 978 MoBa mothers had returned the 8-year MoBa follow-up questionnaire. Means and standard deviations for a range of socio-demographic characteristics, estimated in the three samples, are presented, with −log_10_  *P* values for pairwise differences in means (Z-tests) and variances (F-tests) between the samples, in Table 1. Overall, differences between the sample and the target population reflected a healthy–wealthy volunteer bias. Most notably, participating mothers demonstrated higher educational attainment, lower unemployment rates, and lower prevalences of internalizing disorders compared with the target population.

**Table 1:** MoBa baseline and follow-up sample, means, and standard deviations, with –log_10_ P-values from Z-tests for mean and F-tests for variance differences between target population and sample values.

		Target population (*N* = 296 987)		Baseline sample (*N* = 86 154)				Follow-up sample (*N* = 36 978)					
Who	Variable	Mean^a^	SD^a^	Mean	SD	*Z*-test (population)	*F*-test (population)	Mean	SD	*Z*-test (population)	*F*-test (population)	*Z*-test (baseline)	*F*-test (baseline)
Mothers	Age (years)	29.99	5.22	30.58	4.62	229	416	31.1	4.46	424	334	76	16
	Birthplace Norway^a^	0.83	0.001	0.92	0.001	1340	–	0.93	0.001	1015	–	11	–
	Civil status (married)^a^	0.44	0.001	0.45	0.002	13	–			57	–	21	–
	University degree^a^	0.46	0.001	0.62	0.001	1605	–	0.7	0.001	1928	–	160	–
	Education (years)	14.71	3.01	15.75	2.52	2203	876	16.23	2.27	2620	986	227	117
	Household income (Norwegian Kronet)	744 412	34 487	744 551	45 396	16	2407	744 636	68 142	9	9285	1.6	2020
	Employment status (unemployed)^a^	0.2	0.001	0.11	0.001	1005	–	0.08	0.001	1065	–	45	–
	Hours worked	25.96	12.29	28.28	11.81	549	45	30.05	10.68	971	261	146	112
	Residence settlement population density	13.73	1.71	13.68	1.75	15	22	13.7	1.76	2.7	17	1.6	0.8
	Internalizing (probable diagnosis)^a^	0.36	0.001	0.31	0.001	142	–	0.27	0.001	101	–	22	–
	ADHD (probable diagnosis)^a^	0.03	0.001	0.02	0.001	33	–	0.02	0.001	264	–	45	–
Children	Year of birth	2004.6	2.31	2005	2.15	472	142	2005.4	1.82	1237	737	250	315
	Sex (male)^a^	0.51	0.001	0.52	0.001	1.5	–	0.51	0.001	0.5	–	1.6	–
	Number of Norwegian parents	1.32	0.64	1.14	0.41	1936	5006	1.12	0.36	1548	3405	16.8	131
	National test score (average)	−0.02	0.88	0.15	0.86	630	16	0.27	0.85	890	22	123.3	3.6
	Internalizing (probable diagnosis)^a^	0.12	0.001	0.11	0.001	18	–	0.10	0.001	53	–	14	–
	ADHD (probable diagnosis)^a^	0.09	0.001	0.08	0.001	18	–	0.06	0.001	50	–	13	–

a Proportions and errors – for which F-tests are not applicable. See Supplementary file 4 for full test statistics.

### Bias quantification and correction

#### Sample representativeness: mean values

**Figure 4:**
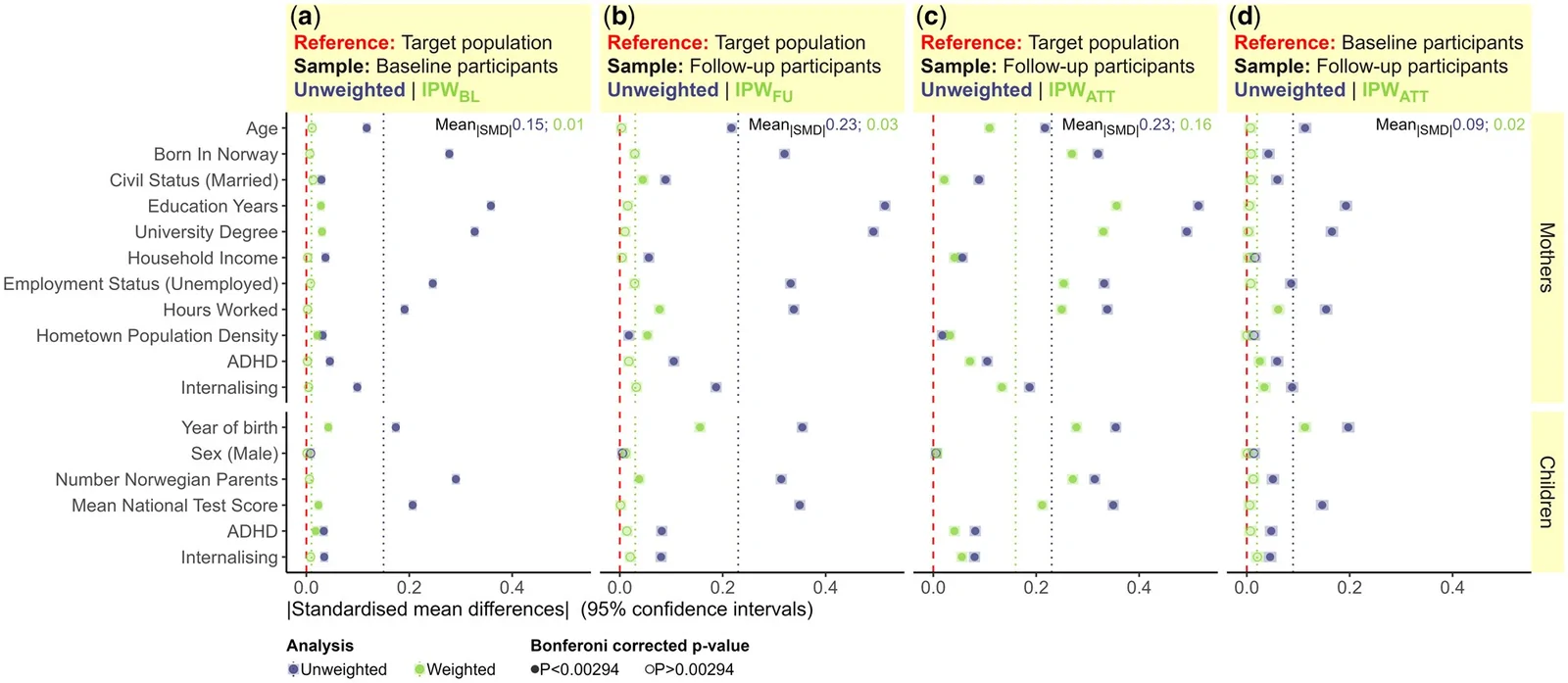
Standardized mean differences (Cohen’s d or Cohen’s g; points) and 95% confidence intervals (shading) between reference values (reference; dashed line) and unweighted and weighted sample estimates. The reference set, stratified sample, and corresponding weight are labelled in the panel headers. For each comparison set, unweighted estimates and weighted estimates are plotted. Dotted lines represent the average standardized mean difference (mean|SMD|) in the unweighted and weighted analysis sets, and the corresponding value is labelled in the top-right corner. The three IPW here were those estimated from the prediction of participation at (a) baseline (BL) and (b) follow-up (FU); (c, d) the prediction of attrition (ATT).

Figure 4 displays standardized mean differences, where unweighted and weighted sample estimates are compared with the corresponding target-population reference values. Compared with the population, the unweighted estimates in the baseline sample were biased considerably (mean_|SMD|_ = 0.15). The weighted estimates were substantially closer to the population values (0.01), where *IPW*_BL_ reduced the bias by 93%. The unweighted estimates in the follow-up sample were biased further (0.23) and the weighted estimates were substantially closer to the population values (0.03), where *IPW*_FU_ reduced the bias by 87%. However, the *IPW*_ATT_-weighted estimates were still considerably biased away from the population values (0.16). When the follow-up and baseline sample estimates were compared, the average difference in the unweighted analyses was 0.09 and the *IPW*_ATT_-weighted analyses reduced this difference to 0.02 (78% reduction).

#### Estimate representativeness: outcome–exposure associations

**Figure 5:**
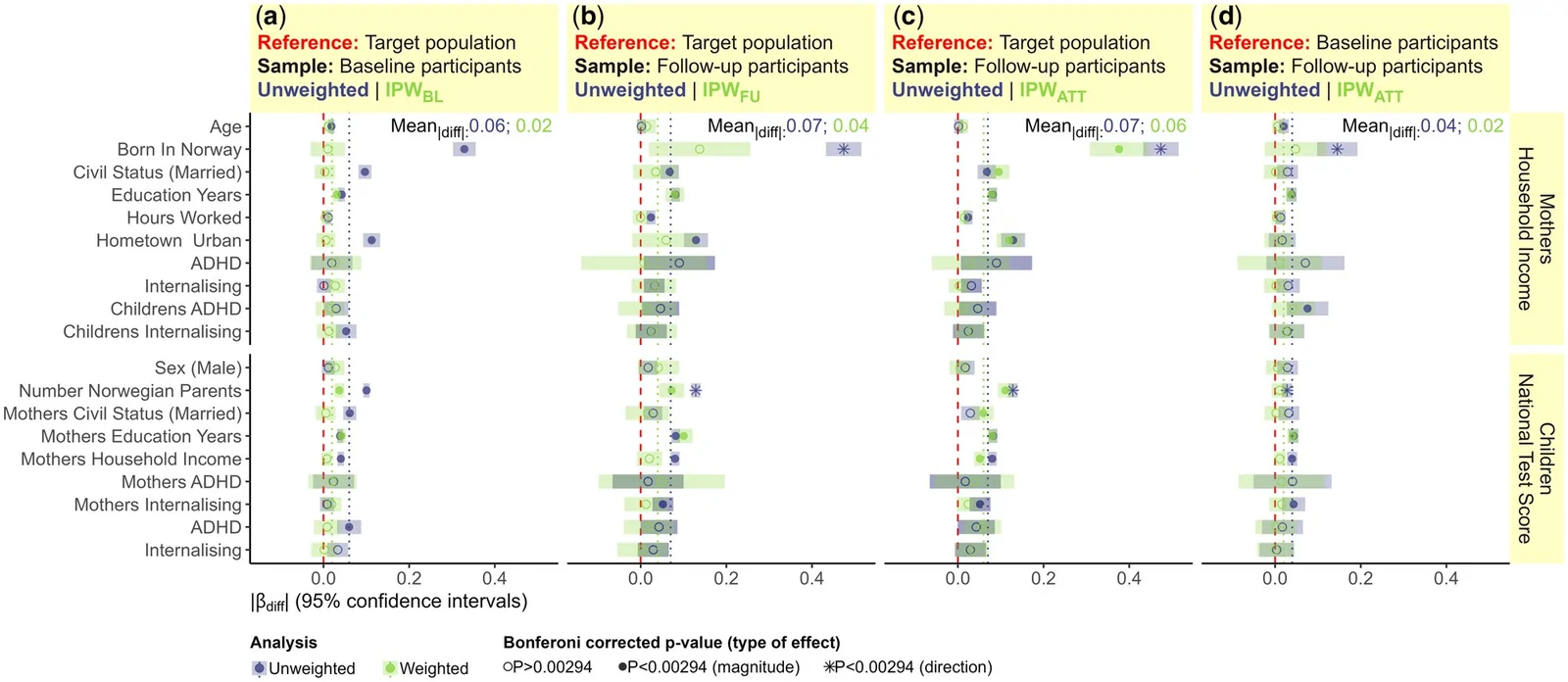
Univariable regression effect size differences (points) and 95% confidence intervals (shading) between reference values (dashed line) and unweighted and weighted sample estimates for two outcomes—(i) mothers’ household income (top panel) and (ii) children’s national test scores (bottom panel)—and nine covariates (*y*-axis). The reference set, stratified sample, and corresponding weight are labelled in the panel headers. For each comparison set, unweighted and weighted estimates are plotted. Dotted lines represent the average effect size difference (mean|diff|) in the unweighted and weighted analysis sets, and the corresponding value is labelled in the top-right corner. The three IPW here were those estimated from the prediction of participation at (a) baseline (BL) and (b) follow-up (FU); (c, d) the prediction of attrition (ATT).

Figure 5 displays the absolute association effect size differences following the univariable linear regression analyses. We compared the unweighted and weighted effect estimates from each MoBa sample (baseline and follow-up) directly to the true estimates derived from the target population. When compared with the population estimates, the unweighted estimates in the baseline sample were biased (mean_|diff|_ = 0.08). The weighted estimates were closer to the population values (0.02), where *IPW*_BL_ reduced the bias by 75%. When compared with the population estimates, the unweighted estimates of univariable effect size in the follow-up sample were also biased (0.07). *IPW*_FU_ reduced the bias by 43% (0.04) and *IPW*_ATT_ reduced the bias by 14% (0.06). When the follow-up sample was compared with the baseline sample coefficients, the extent of the bias was small (0.04) and *IPW*_ATT_-weighted analyses reduced this difference to 0.02 (50%).

Most of the association effect size differences were due to the magnitude of the effect but 4 estimates (out of 57 unweighted tests) had a different direction of effect. The weighted analyses were sufficient in that all estimated associations were in the same direction as the population value. For example, the effect of being born in Norway on mothers’ household income was *β* = 0.46 (SE = 0.005) in the population, *β* = −0.02 (SE = 0.021) in the unweighted follow-up sample, *β* = 0.32 (SE = 0.06) for *IPW*_FU_ analyses, and *β* = 0.08 (SE = 0.04) for *IPW*_ATT_ analyses.

## Discussion

In this study, we used Norwegian population registry data to model the probability of baseline, follow-up, and continued participation in the MoBa Study to compute IPW. We quantified bias from selective participation and attrition in participant socio-demographics and exposure–outcome associations. We then compared the effectiveness of multiple sampling weights in recovering the representative estimates.

Differences between MoBa and the population were consistent with healthy–wealthy volunteer bias as demonstrated in other studies [3, 14]. As noted, participating mothers generally exhibited higher socio-economic status and better mental health. The largest effect sizes for mean differences involved education and occupational variables. However, across the variable set, the mean absolute effect size of the selective participation was small (Cohen’s d = 0.15, 0.1 SD) [22].

While differences between the population and the sample existed for most mean estimates, some univariable associations (including non-representative variables) were representative of the population. For example, differences existed for mothers’ household income and the prevalences of internalizing disorders, yet the association between them was representative. Similar findings have been reported elsewhere to support the accuracy of estimates from selected samples [15]. Nonetheless, some associations showed substantial bias. For example, the association between being born in Norway and household income was considerably smaller in the baseline sample compared with that in the target population (*β*_Pop_ = 0.46, SE_Pop_ = 0.005; *β*_BL_ = 0.13, SE_BL_ = 0.013; *Z*_DIFF_ = 24.46).

In our study, the non-representative estimates of association primarily stemmed from differences in the magnitude of effect rather than directional differences. While our findings suggest that selection bias does not have a large impact on the substantive interpretations of exposure–outcome associations, these examples warrant caution. Studies should assess and report the impacts of selective participation on all analysis variables and use methods to account for selection bias when reporting effect sizes.

Overall, sampling weights substantially reduced the differences between the target population and the study sample. Inverse probability of continued-participation weights were relatively effective at reducing attrition bias but, as expected, were less effective at recovering population values due to selective baseline participation. Using attrition weights is often the best option available, as the data required to compute baseline participation weights are rare. This approach is important in the context of longitudinal analysis, in which biases due to attrition threaten internal validity. However, using attrition weights for external validity (generalizability) depends on a representative baseline sample, which our study, and previous studies, lack [3].

Demonstrating potential uses of population-wide, individual-level data is a major strength of this study. However, limitations exist: our sampling weights, computed primarily from socio-demographic variables, may not effectively account for selection bias in other domains (e.g. ‘clinical variables’); key predictors of participation may be missing from our models; missing data in the prediction variable set were imputed by using single imputation—for which the limitations are well documented [23]. Therefore, we must stress that our analyses serve only to assess the impact of representativeness and weighting in this sample. Furthermore, the attrition weight variables were recorded at baseline, years before dropout; a more effective weight could use time-varying covariates closer to dropout. Relatedly, when estimating weights for the follow-up sample analysis, we utilized a single-step model, *IPW*_FU_. While this performed well in our analyses, it should be noted that baseline participation and subsequent attrition are distinct selection mechanisms driven by different determinants. Thus, the standard approach in many settings is to specify separate selection models for each stage and multiply the corresponding weights (*IPW*_BL_ × *IPW*_ATT_). Relying solely on a single-step weight may not substantially reduce bias in other cohorts, particularly when time-varying covariates associated with attrition are available. This is a common and challenging limitation for studies with selective attrition. Our conclusions may be limited to the socio-demographic and mental health variables under investigation and the associations observed in this sample may not be generalizable to samples with different patterns of sample selection.

Numerous studies have estimated the sampling weights for observational cohorts [9, 14, 16, 24, 25]. Cohort study teams should prioritize researcher access to sampling weights and provide guidelines for their usage. We have shared our analysis pipeline so as to support these efforts. However, the efficacy of participation weights relies on target-population datasets, which are rare. Previous work has shown the effectiveness of summary population-level data for correcting sample estimates via direct standardization [11]. Future work should compare adjustments that use individual-level population-wide registry data with those using publicly available summary data, within the same sample (e.g. [12]). If weights from summary data are effective, then this might be a more achievable strategy for many population-based cohort studies. However, direct standardization approaches rely on having target-population statistics on all of the covariates relevant for the outcome and selection models [12].

Ultimately, ensuring representativeness in observational cohort analyses is critical for translating research findings into healthcare advances that benefit those in greatest need. Improving representativeness and assessing the impacts of selection are critical for minimizing wasted resources spent pursuing erroneous associations. While the bias observed in this study may not necessarily be of sufficient magnitude to substantively impact exposure–outcome associations, some differences were concerning. Several previous studies have highlighted that sample non-representativeness does not always give rise to estimate non-representativeness [6, 15]. However, referencing previous studies does not provide evidence for the generalizability of new findings. Associations from studies without the investigation and reporting of sample and effect representativeness should be interpreted cautiously.

## Ethics approval

Ethical approval for this work has been given by the Regional Committees for Medical and Health Research Ethics (REK; 2017/2205).

## Supplementary Material

dyag122_Supplementary_Data

## Data Availability

Data from MoBa and the Medical Birth Registry of Norway used in this study are managed by the national health register holders in Norway (Norwegian Institute of Public Health) and can be made available to researchers, provided there is approval from the Regional Committees for Medical and Health Research Ethics (REC), compliance with the EU General Data Protection Regulation (GDPR), and approval from the data owners. The consent given by the participants does not open up the storage of data on an individual level in repositories or journals. Researchers who want access to datasets for replication should apply through helsedata.no. Access to datasets requires approval from the Regional Committee for Medical and Health Research Ethics in Norway and an agreement with MoBa.
