# Supplementary material for: Quantifying and adjusting for selection biases in the Norwegian Mother, Father and Child Cohort Study using population-wide individual-level registry information
Source: Int J Epidemiol. 2026 Jul 25;55(4):dyag122. doi: 10.1093/ije/dyag122 (PMC13401474; doi:10.1093/ije/dyag122)
Supplement: dyag122_Supplementary_Data [file dyag122_supplementary_data.zip › ije-2025-04-0715-File008.docx]

Quantifying and adjusting for selection biases in the Norwegian Mother, Father and Child Cohort Study using population-wide individual-level registry information

Supplementary file 1

[**Samples 2**](#_2he1w6alapk1)

[Population reference set (Eligible to participate pregnancies) 2](#_w3h1nlgybei8)

[Figure S1. Eligibility and participation by geographical region. 3](#_2ilr9d7p2tcm)

[Table S1. Participation rates of Norwegian municipalities 4](#_stki3emm8x6u)

#

## Samples

### Population reference set (Eligible to participate pregnancies)

The population reference set was drawn from SSB data, a national register containing information about all individuals in Norway. All pregnancies with delivery dates between 07/1999 and 07/2009 (N=700,615) were considered as eligible pregnancies and used to identify parents in the population that were likely to have been invited to take part in MoBa. Pregnancies were restricted to those who were born in Norway (N=575,746) with the exception of those known participants of MoBa who were not born in Norway (N=145). Eligibility for each pregnancy in the data was further refined by computing geographically linked recruitment windows. Mother’s geographical location of residence at the time of birth was based on municipality and district variables, which were obtained from [SSB](https://www.ssb.no/statbank/table/06198) and joined to the MoBa data by merging on Mothers ID and the child’s date of birth. Municipality and district were combined to create a 6-digit geographical code (geo-code). Geo-codes for some locations have changed over time, thus all codes were harmonised to the most recent coding scheme (2020) using data from [SSB](https://www.ssb.no/klass/klassifikasjoner/131/versjon/1846). After harmonization, pregnancies were grouped by their geo-code. With the exception of known MoBa participants (N=566), pregnancies where neither parent had a recorded geo-code were excluded (N_excl_=6,108; N=569,101). There were 1,695 unique geo-codes in the data. For each geo-code the recruitment start date was determined by the oldest MoBa child participant’s birthdate (MM/YYYY) and the end date was the youngest MoBa child participant’s birthday (see Figure S5 for distribution of participation across Norway and geo-code recruitment windows). Only children with birthdays within this window were retained as (likely) eligible to participate. If there were no MoBa participants in the geo-code, then all pregnancies were excluded (N_excl_=153,820), as we assume mothers in this geo-code were never invited (e.g. no participating hospitals in the geo-code). At this point there were 415,303 eligible pregnancies. Some mothers had multiple eligible pregnancies. Mothers were restricted to 1 pregnancy in the data and participating pregnancies were prioritized. Where mothers participated (or not) with multiple pregnancies, the first pregnancy was selected (based on the earliest delivery date). As such, the population reference set included 296,987 pregnancies.

| **Figure S1. Eligibility and participation by geographical region.** **A.** Proportion of mothers from each municipality who initially participated in the MoBa study; **B.** Proportion of the MoBa mothers in the study (Q1) coming from each municipality; **C.** Recruitment window size in months; **D.** Recruitment windows (start-dates to end dates) | |
| --- | --- |
| ***A***  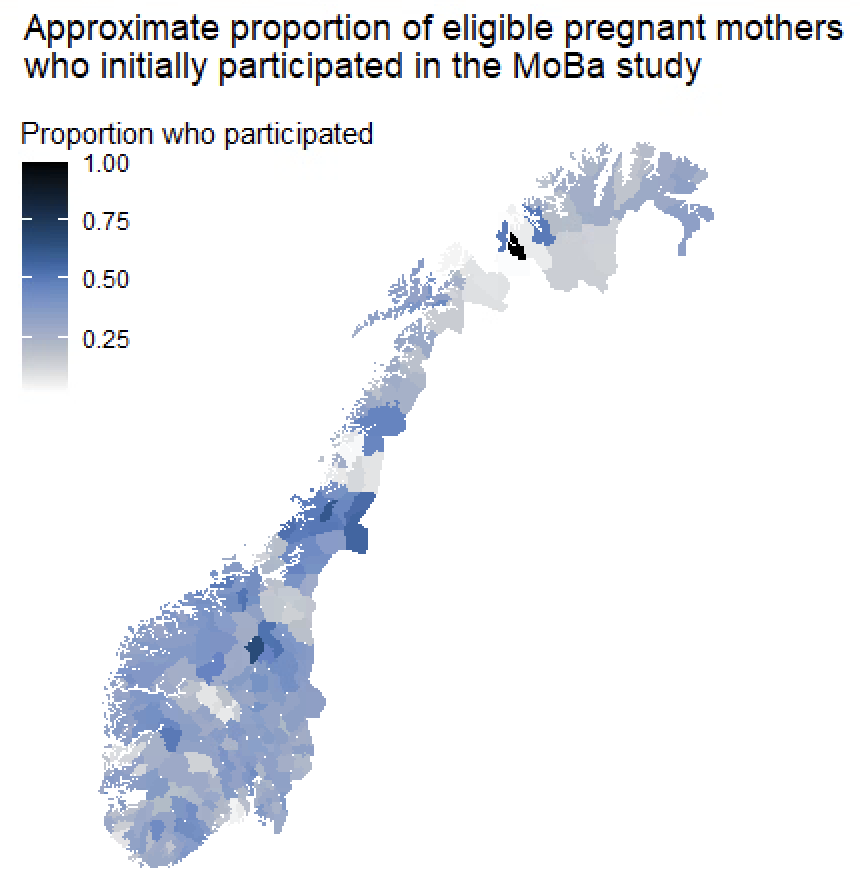 | ***B***  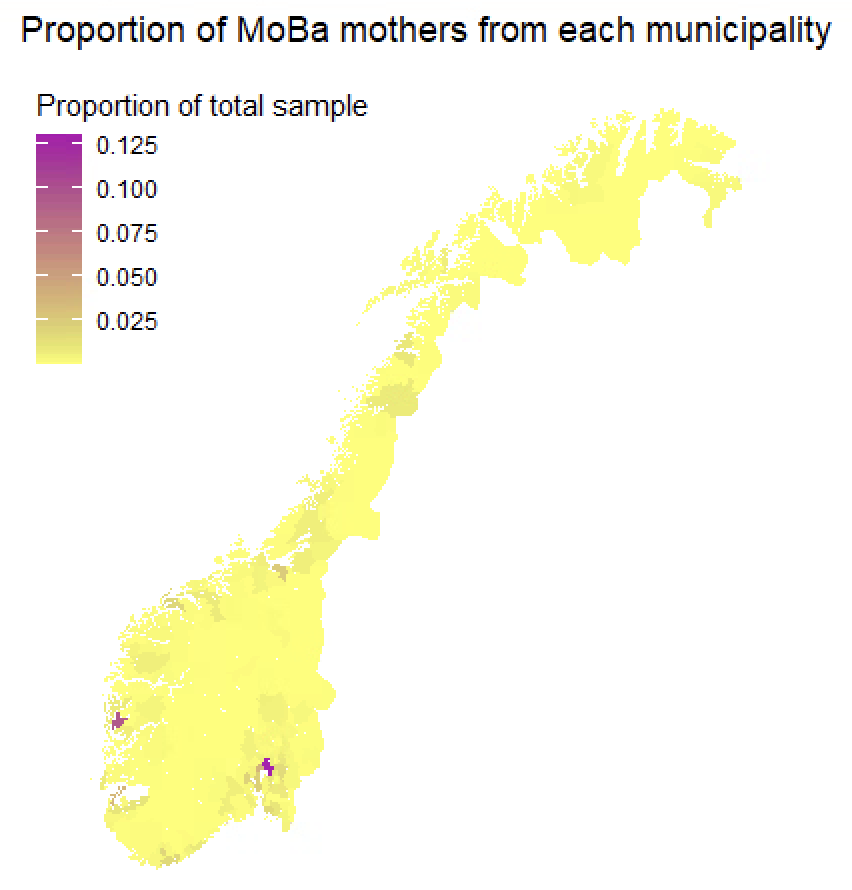 |
| ***C*** 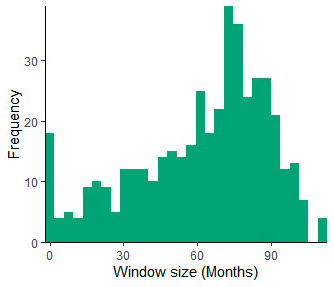 | ***D***  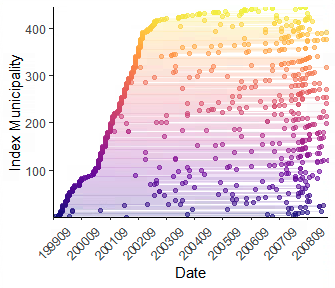 |

### **Table S1.** Participation rates of Norwegian municipalities

(**A)** ranked by the **number*** of mothers who initially participated (returned Q1) in the MoBa study and (**B)** ranked by **lowest proportion*** of participating mothers

| ***A (Top 20 for N)***  **Location** | **Recruitment window (months)** | **Number  Participated^A^** | **Proportion Participated** | **Sample  Proportion (Q1)** |
| --- | --- | --- | --- | --- |
| Oslo-Oslove | 81 | 10995 | 0.23 | 0.131 |
| Bergen | 110 | 7881 | 0.38 | 0.094 |
| Bærum | 102 | 3254 | 0.43 | 0.039 |
| Stavanger | 90.9 | 2932 | 0.3 | 0.035 |
| Trondheim | 92.1 | 2176 | 0.19 | 0.026 |
| Asker | 91.8 | 1928 | 0.4 | 0.023 |
| Drammen | 75 | 1632 | 0.32 | 0.019 |
| Kristiansand | 75.9 | 1507 | 0.29 | 0.018 |
| Lillestrøm | 90 | 1527 | 0.32 | 0.018 |
| Ålesund | 101 | 1522 | 0.38 | 0.018 |

***B (bottom 10 for proportion)***

| Tromsø | 30.9 | 45 | 0.01 | 0.001 |
| --- | --- | --- | --- | --- |
| Storfjord-Omasvuotna- Omasvuono | 30.9 | <10 | 0.02 | <0.001 |
| Vefsn | 81 | 19 | 0.03 | <0.001 |
| Senja | 30.8 | 21 | 0.04 | <0.001 |
| Larvik | 26.9 | 58 | 0.05 | 0.001 |
| Skjervøy | <1 | <10 | 0.05 | <0.001 |
| Nordreisa | <1 | <10 | 0.06 | <0.001 |
| Sandefjord | 81 | 130 | 0.07 | 0.002 |
| Nord-Aurdal | 56.8 | 20 | 0.07 | <0.001 |
| Brønnøy | 32.9 | 23 | 0.07 | <0.001 |

### 
