# Supplementary material for: Quantifying and adjusting for selection biases in the Norwegian Mother, Father and Child Cohort Study using population-wide individual-level registry information
Source: Int J Epidemiol. 2026 Jul 25;55(4):dyag122. doi: 10.1093/ije/dyag122 (PMC13401474; doi:10.1093/ije/dyag122)
Supplement: dyag122_Supplementary_Data [file dyag122_supplementary_data.zip › ije-2025-04-0715-File009.docx]

Quantifying and adjusting for selection biases in the Norwegian Mother, Father and Child Cohort Study using population-wide individual-level registry information

Supplementary file 2

[Variables 2](#_Toc233533893)

[Missing data 2](#_Toc233533894)

[Statistisk Sentralbyrå (SSB) variables 3](#_Toc233533895)

[**Table S2.** Full list of variables obtained from Statistisk Sentralbyrå dataset, used in the prediction models of participation 3](#_Toc233533896)

[SSB dropped categories 38](#_Toc233533897)

[Norwegian Education Registry data: National test scores 38](#_Toc233533898)

[Figure S 2. Distributions of national test scores across Mathematics and English. 38](#_Toc233533899)

[Norwegian Control and Payment of Health Reimbursements Database (KUHR): Diagnostic codes 39](#_Toc233533900)

[References 39](#_Toc233533901)

## Variables

### Missing data

Where we had incomplete data on predictors of participation (Table S2), we performed single imputation of missing values using random forest regression and classification models using [mlim](https://cran.r-project.org/web/packages/mlim/mlim.pdf) [^1^](https://paperpile.com/c/ayHOwg/r3F6). We used the mlim::mlim.preimpute function with its default settings, which implements a multivariate iterative imputation procedure based on Random Forest, leveraging the high-performance missRanger package [^2^](https://paperpile.com/c/ayHOwg/qH2N), based on the missForest algorithm [^3^](https://paperpile.com/c/ayHOwg/JC18). First, all missing values in the dataset are filled with a preliminary estimate (e.g., mean for continuous, mode for categorical). The algorithm then iteratively models each variable containing missing data as a dependent variable, using all other variables in the dataset as predictors. For each variable, a Random Forest model is trained on the subset of observed values and is then used to predict its missing values. These predictions replace the initially imputed values. This cycle is repeated for all variables over several iterations until the imputed values stabilise (i.e., the difference between imputation steps falls below a convergence threshold), resulting in a single completed dataset for analysis. We opted for single imputation of missing values, because of the large sizes of the prediction datasets (N=296,987; predictors≈220), and complexities associated with pooling estimates across the downstream analyses. Single imputation allows us to estimate one prediction model per participation outcome, to derive a single weight, which can be applied once in each downstream analysis to demonstrate the impact of selection bias on the MoBa sample, to highlight the issue of selection bias to researchers using this dataset.

While single imputation provides a complete dataset for analysis and avoids the biases associated with listwise deletion, it has notable limitations. Simple methods for handling missing covariates can be highly sensitive to model misspecification and may lead to biased parameter estimates and invalid conclusions [^4^](https://paperpile.com/c/ayHOwg/jTvE). Single imputation replaces each missing value with only one estimate, failing to account for the statistical uncertainty in the imputation process [^5^](https://paperpile.com/c/ayHOwg/NGEM). As such, subsequent analyses may underestimate the standard errors of parameter estimates, leading to overly narrow confidence intervals and potentially inflated Type I error rates [^6^](https://paperpile.com/c/ayHOwg/ZVIJ). Furthermore, while Random Forest is a flexible non-parametric method, its performance degrades if the missing data mechanism is Missing Not At Random (MNAR) and not accounted for in the model [^3^](https://paperpile.com/c/ayHOwg/JC18). For a more robust approach that includes imputation uncertainty into final estimates, Multiple Imputation (MI) is recommended. By generating m>1 imputed datasets and pooling the final estimates (means, outcome exposure associations using Rubin's rules [^7^](https://paperpile.com/c/ayHOwg/0aMZ), standard errors and confidence intervals better reflect the uncertainty due to missing data. Despite these limitations, single imputation was deemed appropriate for the current study because our primary objective is descriptive—specifically, to assess the impact of representativeness and weighting—rather than to estimate causal effects where precise standard errors for hypothesis testing are critical.

###

### Statistisk Sentralbyrå (SSB) variables

#### **Table S2.** Full list of variables obtained from Statistisk Sentralbyrå dataset, used in the prediction models of participation

**Dimensions:** 296987 x 110

| No | Variable | Stats / Values | Freqs (% of Valid) | Graph | Valid | Missing |
| --- | --- | --- | --- | --- | --- | --- |
| 1 | Child Sex [factor] | 1. 1 2. 2 | 151406 (51.2%) 144371 (48.8%) | 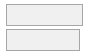 | 295777 (99.6%) | 1210 (0.4%) |
| 2 | Child DOB [integer] | Mean (sd) : 200465.1 (231.14) min < med < max: 199909 < 200501 < 200908 IQR (CV) : 308 (0) | 120 distinct values | 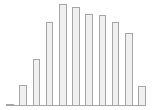 | 295777 (99.6%) | 1210 (0.4%) |
| 3 | Father Age [numeric] | Mean (sd) : 32.99 (6.22) min < med < max: 13.58 < 32.5 < 75.92 IQR (CV) : 7.75 (0.19) | 638 distinct values | 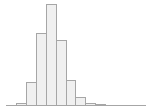 | 295765 (99.6%) | 1222 (0.4%) |
| 4 | Mother Age [numeric] | Mean (sd) : 29.98 (5.23) min < med < max: 14.08 < 30 < 53.67 IQR (CV) : 7.25 (0.17) | 419 distinct values | 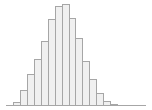 | 295776 (99.6%) | 1211 (0.4%) |
| 5 | Parental Age Gap [numeric] | Mean (sd) : 3.01 (4.72) min < med < max: -25.25 < 2.33 < 46.67 IQR (CV) : 5 (1.57) | 862 distinct values | 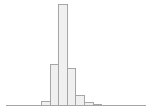 | 295765 (99.6%) | 1222 (0.4%) |
| 6 | Child Died [factor] | 1. 0 2. 1 | 294309 (99.5%) 1468 (0.5%) | 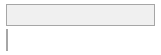 | 295777 (99.6%) | 1210 (0.4%) |
| 7 | Mother Died [factor] | 1. 0 2. 1 | 293039 (99.1%) 2738 (0.9%) | 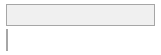 | 295777 (99.6%) | 1210 (0.4%) |
| 8 | Mother Mother Died [factor] | 1. 0 2. 1 | 243320 (82.3%) 52457 (17.7%) | 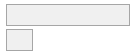 | 295777 (99.6%) | 1210 (0.4%) |
| 9 | Mother Father Died [factor] | 1. 0 2. 1 | 205510 (69.5%) 90267 (30.5%) | 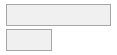 | 295777 (99.6%) | 1210 (0.4%) |
| 10 | Father Died [factor] | 1. 0 2. 1 | 289342 (97.8%) 6435 (2.2%) | 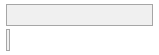 | 295777 (99.6%) | 1210 (0.4%) |
| 11 | Father Mother Died [factor] | 1. 0 2. 1 | 225427 (76.2%) 70350 (23.8%) | 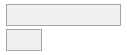 | 295777 (99.6%) | 1210 (0.4%) |
| 12 | Father Father Died [factor] | 1. 0 2. 1 | 184149 (62.3%) 111628 (37.7%) | 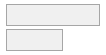 | 295777 (99.6%) | 1210 (0.4%) |
| 13 | Child Number Norwegian Parents [factor] | 1. 2 2. 1 3. 0 | 230978 (78.1%) 36001 (12.2%) 28798 (9.7%) | 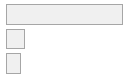 | 295777 (99.6%) | 1210 (0.4%) |
| 14 | Mother Number Norwegian Parents [factor] | 1. 2 2. 0 3. 1 | 236209 (79.9%) 47903 (16.2%) 11664 (3.9%) | 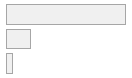 | 295776 (99.6%) | 1211 (0.4%) |
| 15 | Father Number Norwegian Parents [factor] | 1. 2 2. 0 3. 1 | 238922 (80.8%) 44979 (15.2%) 11864 (4.0%) | 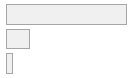 | 295765 (99.6%) | 1222 (0.4%) |
| 16 | Mother Hours Worked [numeric] | Mean (sd) : 27.45 (12.99) min < med < max: 1 < 35.5 < 99 IQR (CV) : 19.5 (0.47) | 4108 distinct values | 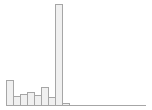 | 243981 (82.2%) | 53006 (17.8%) |
| 17 | Father Hours Worked [numeric] | Mean (sd) : 33.45 (10.43) min < med < max: 1 < 37.5 < 99 IQR (CV) : 0 (0.31) | 2770 distinct values | 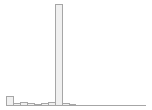 | 267657 (90.1%) | 29330 (9.9%) |
| 18 | Mother Professional Income [numeric] | Mean (sd) : 6.87 (0) min < med < max: 6.87 < 6.87 < 6.89 IQR (CV) : 0 (0) | 265955 distinct values | 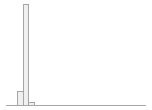 | 295721 (99.6%) | 1266 (0.4%) |
| 19 | Mother Salary Income [numeric] | Mean (sd) : 4.46 (0.07) min < med < max: 4.24 < 4.47 < 5.19 IQR (CV) : 0.1 (0.02) | 262090 distinct values | 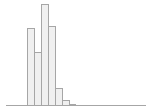 | 295721 (99.6%) | 1266 (0.4%) |
| 20 | Mother Net Business Income [numeric] | Mean (sd) : 6.87 (0) min < med < max: 6.87 < 6.87 < 6.89 IQR (CV) : 0 (0) | 18196 distinct values | 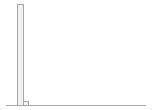 | 295721 (99.6%) | 1266 (0.4%) |
| 21 | Mother Capital Income [numeric] | Mean (sd) : 6.84 (0) min < med < max: 6.83 < 6.84 < 6.9 IQR (CV) : 0 (0) | 123662 distinct values | 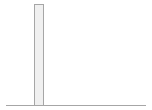 | 295721 (99.6%) | 1266 (0.4%) |
| 22 | Mother Interest Income [numeric] | Mean (sd) : 3.6 (0.02) min < med < max: 3.59 < 3.59 < 4.86 IQR (CV) : 0.01 (0.01) | 120818 distinct values | 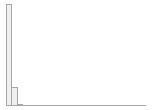 | 295721 (99.6%) | 1266 (0.4%) |
| 23 | Mother Stock Dividends [numeric] | Mean (sd) : 0.07 (0.36) min < med < max: 0 < 0 < 5.86 IQR (CV) : 0 (5.05) | 7786 distinct values | 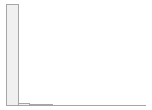 | 295721 (99.6%) | 1266 (0.4%) |
| 24 | Mother Realization Gains [numeric] | Mean (sd) : 3.17 (0.03) min < med < max: 3.11 < 3.17 < 4.54 IQR (CV) : 0 (0.01) | 6099 distinct values | 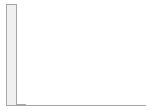 | 295721 (99.6%) | 1266 (0.4%) |
| 25 | Mother Realization Loss [numeric] | Mean (sd) : 2.75 (0.03) min < med < max: 1.83 < 2.75 < 4.98 IQR (CV) : 0 (0.01) | 4228 distinct values | 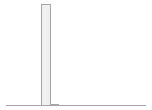 | 295721 (99.6%) | 1266 (0.4%) |
| 26 | Mother Other Capital Income [numeric] | Mean (sd) : 4.33 (0.01) min < med < max: 4.33 < 4.33 < 5.17 IQR (CV) : 0 (0) | 11890 distinct values | 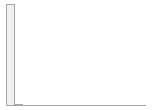 | 295721 (99.6%) | 1266 (0.4%) |
| 27 | Mother Transfers [numeric] | Mean (sd) : 4.48 (0.02) min < med < max: 4.37 < 4.47 < 4.7 IQR (CV) : 0.02 (0.01) | 179641 distinct values | 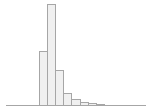 | 295721 (99.6%) | 1266 (0.4%) |
| 28 | Mother Taxable Transfers [numeric] | Mean (sd) : 4.47 (0.02) min < med < max: 4.38 < 4.46 < 4.69 IQR (CV) : 0 (0) | 57831 distinct values | 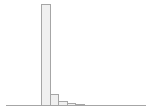 | 295721 (99.6%) | 1266 (0.4%) |
| 29 | Mother Pensions From The National Insurance [numeric] | Mean (sd) : 4.47 (0.01) min < med < max: 4.38 < 4.46 < 4.62 IQR (CV) : 0 (0) | 27625 distinct values | 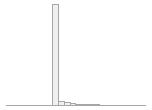 | 295721 (99.6%) | 1266 (0.4%) |
| 30 | Mother Retirement Pensions [numeric] | Mean (sd) : 0 (0.01) min < med < max: 0 < 0 < 2.87 IQR (CV) : 0 (95.15) | 28 distinct values | 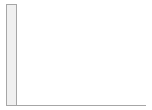 | 295721 (99.6%) | 1266 (0.4%) |
| 31 | Mother Disability Pension [numeric] | Mean (sd) : 0.03 (0.31) min < med < max: 0 < 0 < 4.03 IQR (CV) : 0 (11.07) | 2058 distinct values | 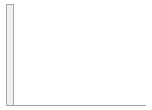 | 295721 (99.6%) | 1266 (0.4%) |
| 32 | Mother Work Settlement Allowance [numeric] | Mean (sd) : 0 (0.03) min < med < max: 0 < 0 < 1.81 IQR (CV) : 0 (34.98) | 220 distinct values | 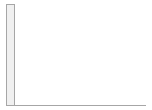 | 295721 (99.6%) | 1266 (0.4%) |
| 33 | Mother Service Pensions [numeric] | Mean (sd) : 3.57 (0.01) min < med < max: 3.55 < 3.57 < 4.15 IQR (CV) : 0 (0) | 3449 distinct values | 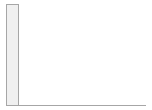 | 295721 (99.6%) | 1266 (0.4%) |
| 34 | Mother Unemployment Benefit [numeric] | Mean (sd) : 0.22 (0.62) min < med < max: 0 < 0 < 3.79 IQR (CV) : 0 (2.81) | 22446 distinct values | 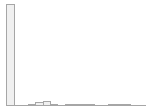 | 295721 (99.6%) | 1266 (0.4%) |
| 35 | Mother Sick Pay [numeric] | Mean (sd) : 4.16 (0.01) min < med < max: 2.87 < 4.15 < 4.38 IQR (CV) : 0 (0) | 72038 distinct values | 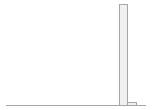 | 295721 (99.6%) | 1266 (0.4%) |
| 36 | Mother Parental Allowance [numeric] | Mean (sd) : 3.56 (0.1) min < med < max: 3.16 < 3.5 < 4.18 IQR (CV) : 0.13 (0.03) | 61727 distinct values | 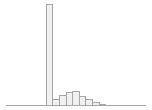 | 295721 (99.6%) | 1266 (0.4%) |
| 37 | Mother Tax Free Transfers [numeric] | Mean (sd) : 3.95 (0.04) min < med < max: 3.88 < 3.94 < 4.43 IQR (CV) : 0.05 (0.01) | 149297 distinct values | 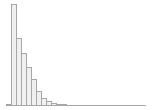 | 295721 (99.6%) | 1266 (0.4%) |
| 38 | Mother Child Benefit [numeric] | Mean (sd) : 3.92 (0.02) min < med < max: 3.88 < 3.91 < 4.14 IQR (CV) : 0.03 (0.01) | 45779 distinct values | 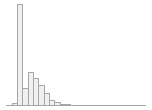 | 295721 (99.6%) | 1266 (0.4%) |
| 39 | Mother Studiestipend [numeric] | Mean (sd) : 2.87 (0.1) min < med < max: 2.83 < 2.84 < 3.68 IQR (CV) : 0 (0.03) | 21087 distinct values | 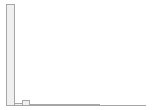 | 295721 (99.6%) | 1266 (0.4%) |
| 40 | Mother Dependent Deduction [numeric] | Mean (sd) : 0.05 (0.2) min < med < max: 0 < 0 < 2.49 IQR (CV) : 0 (4.21) | 383 distinct values | 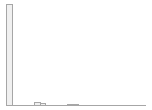 | 295721 (99.6%) | 1266 (0.4%) |
| 41 | Mother Social Assistance [numeric] | Mean (sd) : 0.12 (0.49) min < med < max: 0 < 0 < 4.11 IQR (CV) : 0 (4.05) | 13779 distinct values | 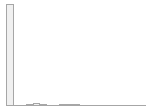 | 295721 (99.6%) | 1266 (0.4%) |
| 42 | Mother Basic And Auxiliary Allowance [numeric] | Mean (sd) : 2.06 (0.07) min < med < max: 2.05 < 2.05 < 3.29 IQR (CV) : 0 (0.04) | 1097 distinct values | 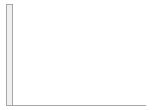 | 295721 (99.6%) | 1266 (0.4%) |
| 43 | Mother Cash Support [numeric] | Mean (sd) : 3.44 (0.04) min < med < max: 3.28 < 3.43 < 3.74 IQR (CV) : 0.04 (0.01) | 25445 distinct values | 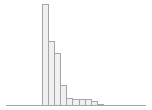 | 295721 (99.6%) | 1266 (0.4%) |
| 44 | Mother Total Income [numeric] | Mean (sd) : 6.86 (0) min < med < max: 6.84 < 6.86 < 6.91 IQR (CV) : 0 (0) | 293796 distinct values | 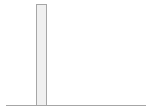 | 295721 (99.6%) | 1266 (0.4%) |
| 45 | Mother Equalized Tax And Negative Transfers [numeric] | Mean (sd) : 4.43 (0.02) min < med < max: 4.38 < 4.43 < 5.55 IQR (CV) : 0.03 (0.01) | 262903 distinct values | 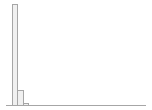 | 295721 (99.6%) | 1266 (0.4%) |
| 46 | Mother Equalized Tax [numeric] | Mean (sd) : 4.43 (0.02) min < med < max: 4.38 < 4.43 < 5.55 IQR (CV) : 0.03 (0.01) | 239789 distinct values | 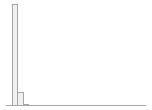 | 295721 (99.6%) | 1266 (0.4%) |
| 47 | Mother Income After Tax [numeric] | Mean (sd) : 6.87 (0) min < med < max: 6.84 < 6.87 < 6.92 IQR (CV) : 0 (0) | 293824 distinct values | 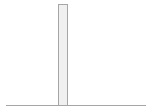 | 295721 (99.6%) | 1266 (0.4%) |
| 48 | Mother Interest Expenses [numeric] | Mean (sd) : 1.84 (1.19) min < med < max: 0 < 2.18 < 4.64 IQR (CV) : 2.2 (0.65) | 189021 distinct values | 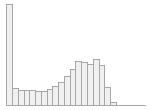 | 295721 (99.6%) | 1266 (0.4%) |
| 49 | Father Professional Income [numeric] | Mean (sd) : 6.87 (0) min < med < max: 6.85 < 6.87 < 7.18 IQR (CV) : 0 (0) | 283538 distinct values | 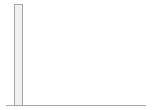 | 294117 (99.0%) | 2870 (1.0%) |
| 50 | Father Salary Income [numeric] | Mean (sd) : 4.52 (0.09) min < med < max: 4.25 < 4.53 < 5.56 IQR (CV) : 0.09 (0.02) | 274584 distinct values | 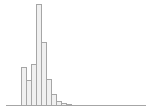 | 294117 (99.0%) | 2870 (1.0%) |
| 51 | Father Net Business Income [numeric] | Mean (sd) : 6.87 (0) min < med < max: 6.85 < 6.87 < 7.18 IQR (CV) : 0 (0) | 45661 distinct values | 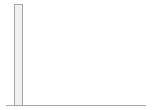 | 294117 (99.0%) | 2870 (1.0%) |
| 52 | Father Capital Income [numeric] | Mean (sd) : 6.84 (0) min < med < max: 6.8 < 6.84 < 7.16 IQR (CV) : 0 (0) | 152753 distinct values | 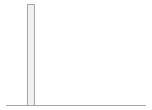 | 294117 (99.0%) | 2870 (1.0%) |
| 53 | Father Interest Income [numeric] | Mean (sd) : 3.6 (0.03) min < med < max: 3.55 < 3.59 < 5.41 IQR (CV) : 0.01 (0.01) | 136028 distinct values | 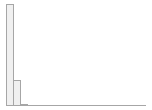 | 294117 (99.0%) | 2870 (1.0%) |
| 54 | Father Stock Dividends [numeric] | Mean (sd) : 0.19 (0.6) min < med < max: 0 < 0 < 6.8 IQR (CV) : 0 (3.09) | 19194 distinct values | 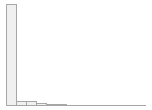 | 294117 (99.0%) | 2870 (1.0%) |
| 55 | Father Realization Gains [numeric] | Mean (sd) : 3.18 (0.07) min < med < max: 3.16 < 3.17 < 5.8 IQR (CV) : 0 (0.02) | 16078 distinct values | 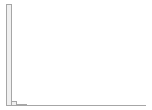 | 294117 (99.0%) | 2870 (1.0%) |
| 56 | Father Realization Loss [numeric] | Mean (sd) : 2.76 (0.09) min < med < max: 2.61 < 2.75 < 5.16 IQR (CV) : 0 (0.03) | 13939 distinct values | 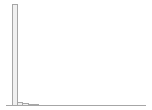 | 294117 (99.0%) | 2870 (1.0%) |
| 57 | Father Other Capital Income [numeric] | Mean (sd) : 4.33 (0.02) min < med < max: 2.89 < 4.33 < 5.66 IQR (CV) : 0 (0) | 21113 distinct values | 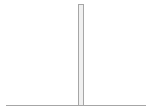 | 294117 (99.0%) | 2870 (1.0%) |
| 58 | Father Transfers [numeric] | Mean (sd) : 4.46 (0.02) min < med < max: 4.23 < 4.45 < 4.82 IQR (CV) : 0 (0) | 68729 distinct values | 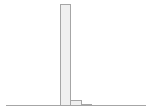 | 294117 (99.0%) | 2870 (1.0%) |
| 59 | Father Taxable Transfers [numeric] | Mean (sd) : 4.47 (0.02) min < med < max: 4.26 < 4.46 < 4.72 IQR (CV) : 0 (0) | 42689 distinct values | 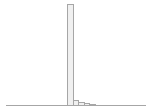 | 294117 (99.0%) | 2870 (1.0%) |
| 60 | Father Pensions From The National Insurance [numeric] | Mean (sd) : 4.47 (0.01) min < med < max: 4.26 < 4.46 < 4.64 IQR (CV) : 0 (0) | 17917 distinct values | 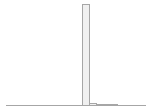 | 294117 (99.0%) | 2870 (1.0%) |
| 61 | Father Retirement Pensions [numeric] | Mean (sd) : 0 (0.04) min < med < max: 0 < 0 < 3.97 IQR (CV) : 0 (91.35) | 42 distinct values | 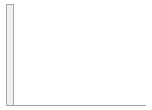 | 294117 (99.0%) | 2870 (1.0%) |
| 62 | Father Disability Pension [numeric] | Mean (sd) : 0.04 (0.35) min < med < max: 0 < 0 < 4.15 IQR (CV) : 0 (9.93) | 2896 distinct values | 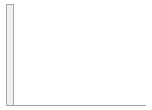 | 294117 (99.0%) | 2870 (1.0%) |
| 63 | Father Work Settlement Allowance [numeric] | Mean (sd) : 0 (0.04) min < med < max: 0 < 0 < 1.35 IQR (CV) : 0 (32.46) | 248 distinct values | 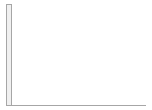 | 294117 (99.0%) | 2870 (1.0%) |
| 64 | Father Service Pensions [numeric] | Mean (sd) : 3.57 (0.01) min < med < max: 3.57 < 3.57 < 4.32 IQR (CV) : 0 (0) | 3518 distinct values | 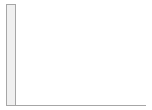 | 294117 (99.0%) | 2870 (1.0%) |
| 65 | Father Unemployment Benefit [numeric] | Mean (sd) : 0.22 (0.64) min < med < max: 0 < 0 < 3.86 IQR (CV) : 0 (2.86) | 23941 distinct values | 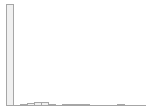 | 294117 (99.0%) | 2870 (1.0%) |
| 66 | Father Sick Pay [numeric] | Mean (sd) : 4.15 (0.01) min < med < max: 4.14 < 4.15 < 4.4 IQR (CV) : 0 (0) | 22123 distinct values | 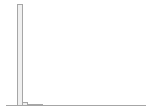 | 294117 (99.0%) | 2870 (1.0%) |
| 67 | Father Parental Allowance [numeric] | Mean (sd) : 3.51 (0.03) min < med < max: 3.5 < 3.5 < 4.05 IQR (CV) : 0.03 (0.01) | 14203 distinct values | 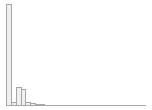 | 294117 (99.0%) | 2870 (1.0%) |
| 68 | Father Tax Free Transfers [numeric] | Mean (sd) : 3.9 (0.03) min < med < max: 3.87 < 3.9 < 4.47 IQR (CV) : 0 (0.01) | 37380 distinct values | 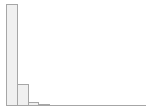 | 294117 (99.0%) | 2870 (1.0%) |
| 69 | Father Child Benefit [numeric] | Mean (sd) : 3.9 (0.01) min < med < max: 3.89 < 3.9 < 4.08 IQR (CV) : 0 (0) | 5709 distinct values | 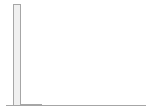 | 294117 (99.0%) | 2870 (1.0%) |
| 70 | Father Studiestipend [numeric] | Mean (sd) : 2.85 (0.05) min < med < max: 2.76 < 2.84 < 3.71 IQR (CV) : 0 (0.02) | 9471 distinct values | 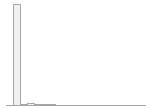 | 294117 (99.0%) | 2870 (1.0%) |
| 71 | Father Dependent Deduction [numeric] | Mean (sd) : 0.04 (0.19) min < med < max: 0 < 0 < 2.53 IQR (CV) : 0 (4.45) | 604 distinct values | 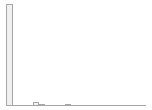 | 294117 (99.0%) | 2870 (1.0%) |
| 72 | Father Social Assistance [numeric] | Mean (sd) : 0.13 (0.56) min < med < max: 0 < 0 < 4.11 IQR (CV) : 0 (4.13) | 14730 distinct values | 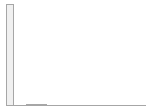 | 294117 (99.0%) | 2870 (1.0%) |
| 73 | Father Basic And Auxiliary Allowance [numeric] | Mean (sd) : 2.06 (0.06) min < med < max: 2.05 < 2.05 < 3.29 IQR (CV) : 0 (0.03) | 927 distinct values | 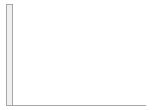 | 294117 (99.0%) | 2870 (1.0%) |
| 74 | Father Cash Support [numeric] | Mean (sd) : 3.41 (0.01) min < med < max: 3.38 < 3.41 < 3.71 IQR (CV) : 0 (0) | 2619 distinct values | 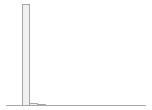 | 294117 (99.0%) | 2870 (1.0%) |
| 75 | Father Total Income [numeric] | Mean (sd) : 6.86 (0) min < med < max: 6.81 < 6.86 < 7.19 IQR (CV) : 0 (0) | 289854 distinct values | 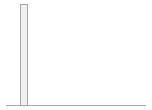 | 294117 (99.0%) | 2870 (1.0%) |
| 76 | Father Equalized Tax And Negative Transfers [numeric] | Mean (sd) : 4.46 (0.04) min < med < max: 4.37 < 4.45 < 6.39 IQR (CV) : 0.04 (0.01) | 285284 distinct values | 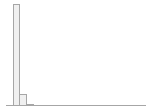 | 294117 (99.0%) | 2870 (1.0%) |
| 77 | Father Equalized Tax [numeric] | Mean (sd) : 4.46 (0.04) min < med < max: 4.37 < 4.45 < 6.39 IQR (CV) : 0.04 (0.01) | 274701 distinct values | 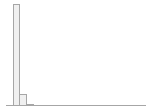 | 294117 (99.0%) | 2870 (1.0%) |
| 78 | Father Income After Tax [numeric] | Mean (sd) : 6.87 (0) min < med < max: 6.82 < 6.87 < 7.17 IQR (CV) : 0 (0) | 289921 distinct values | 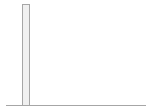 | 294117 (99.0%) | 2870 (1.0%) |
| 79 | Father Interest Expenses [numeric] | Mean (sd) : 2.64 (1.03) min < med < max: 0 < 3.05 < 5.3 IQR (CV) : 1.01 (0.39) | 251861 distinct values | 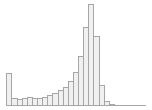 | 294117 (99.0%) | 2870 (1.0%) |
| 80 | Mother Household Income [numeric] | Mean (sd) : 6.87 (0) min < med < max: 6.84 < 6.87 < 7.31 IQR (CV) : 0 (0) | 208144 distinct values | 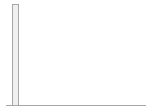 | 232433 (78.3%) | 64554 (21.7%) |
| 81 | Father Household Income [numeric] | Mean (sd) : 6.87 (0) min < med < max: 6.84 < 6.87 < 7.31 IQR (CV) : 0 (0) | 205413 distinct values | 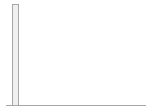 | 232676 (78.3%) | 64311 (21.7%) |
| 82 | Mother Residence Settlement Population Density [numeric] | Mean (sd) : 13.73 (1.71) min < med < max: 11 < 14 < 17 IQR (CV) : 2 (0.12) | 13 distinct values | 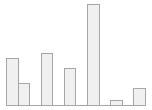 | 296971 (100.0%) | 16 (0.0%) |
| 83 | Mother Residence Settlement Size Km2 [numeric] | Mean (sd) : 65.71 (84.74) min < med < max: 0.02 < 22.9 < 280.3 IQR (CV) : 85.87 (1.29) | 2502 distinct values | 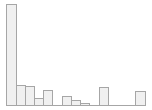 | 293472 (98.8%) | 3515 (1.2%) |
| 84 | Father Residence Settlement Population Density [numeric] | Mean (sd) : 13.7 (1.71) min < med < max: 11 < 14 < 17 IQR (CV) : 3 (0.13) | 13 distinct values | 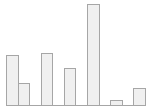 | 296971 (100.0%) | 16 (0.0%) |
| 85 | Father Residence Settlement Size Km2 [numeric] | Mean (sd) : 65.34 (84.37) min < med < max: 0.02 < 22.9 < 280.3 IQR (CV) : 86.07 (1.29) | 2395 distinct values | 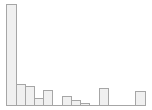 | 295899 (99.6%) | 1088 (0.4%) |
| 86 | Mother Education Code [factor] | 1. Level6 2. Level4 3. Level2 4. Level7 5. Level3 6. Level5 7. Level1 8. Level8 9. Level0 | 104702 (36.7%) 83479 (29.3%) 50444 (17.7%) 26099 (9.2%) 10846 (3.8%) 5117 (1.8%) 2104 (0.7%) 1311 (0.5%) 1046 (0.4%) | 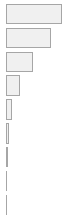 | 285148 (96.0%) | 11839 (4.0%) |
| 87 | Mother Education Years [integer] | Mean (sd) : 14.77 (3.04) min < med < max: 0 < 14 < 22 IQR (CV) : 3 (0.21) | 0 : 1046 (0.4%) 7 : 2104 (0.7%) 10 : 50444 (17.7%) 13 : 10846 (3.8%) 14 : 83479 (29.3%) 15 : 5117 (1.8%) 17 : 104702 (36.7%) 19 : 26099 (9.2%) 22 : 1311 (0.5%) | 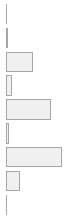 | 285148 (96.0%) | 11839 (4.0%) |
| 88 | Father Education Code [factor] | 1. Level4 2. Level6 3. Level2 4. Level7 5. Level3 6. Level5 7. Level8 8. Level1 9. Level0 | 102608 (35.4%) 68707 (23.7%) 53477 (18.5%) 31647 (10.9%) 14824 (5.1%) 13146 (4.5%) 2812 (1.0%) 1790 (0.6%) 586 (0.2%) | 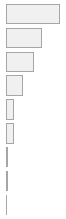 | 289597 (97.5%) | 7390 (2.5%) |
| 89 | Father Education Years [integer] | Mean (sd) : 14.52 (2.99) min < med < max: 0 < 14 < 22 IQR (CV) : 3 (0.21) | 0 : 586 (0.2%) 7 : 1790 (0.6%) 10 : 53477 (18.5%) 13 : 14824 (5.1%) 14 : 102608 (35.4%) 15 : 13146 (4.5%) 17 : 68707 (23.7%) 19 : 31647 (10.9%) 22 : 2812 (1.0%) | 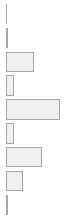 | 289597 (97.5%) | 7390 (2.5%) |
| 90 | Child Birth Place [factor] | 1. Norway 2. Europe 3. Scandinavia 4. Asia 5. Americas 6. Africa | 295657 (100.0%) 46 (0.0%) 33 (0.0%) 24 (0.0%) <10 (0.0%) <10 (0.0%) | 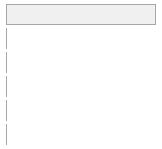 | 295773 (99.6%) | 1214 (0.4%) |
| 91 | Mother Birth Place [factor] | 1. Norway 2. Asia 3. Europe 4. Africa 5. Scandinavia 6. Americas 7. Oceania | 244330 (83.0%) 21005 (7.1%) 12137 (4.1%) 6529 (2.2%) 6268 (2.1%) 4068 (1.4%) 168 (0.1%) | 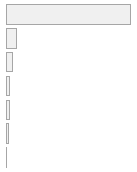 | 294505 (99.2%) | 2482 (0.8%) |
| 92 | Father Birth Place [factor] | 1. Norway 2. Asia 3. Europe 4. Africa 5. Scandinavia 6. Americas 7. Oceania | 247757 (84.2%) 17670 (6.0%) 11263 (3.8%) 7469 (2.5%) 6248 (2.1%) 3580 (1.2%) 347 (0.1%) | 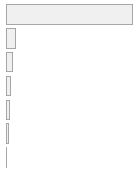 | 294334 (99.1%) | 2653 (0.9%) |
| 93 | Mother Employment Status [factor] | 1. WageEarner 2. OutsideWorkforce 3. Independent 4. Free 5. Qualification | 226171 (76.5%) 58603 (19.8%) 5880 (2.0%) 4387 (1.5%) 656 (0.2%) | 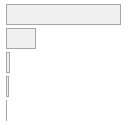 | 295697 (99.6%) | 1290 (0.4%) |
| 94 | Mother Hours Worked Category [factor] | 1. 30plus 2. 19hrs 3. OutsideWorkforce 4. 20_29hrs | 168400 (57.4%) 55419 (18.9%) 38606 (13.2%) 31150 (10.6%) | 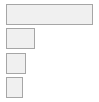 | 293575 (98.9%) | 3412 (1.1%) |
| 95 | Mother Industry Sector1987 [factor] | 1. Private_companies_with_li 2. Municipalities 3. OutsideWorkforce 4. The_State_and_Social_Secu 5. Personal_entrepreneur 6. Private_consumer-oriented 7. State-owned_enterprises 8. County_municipalities 9. Personal_enterprises_etc 10. Independent_municipal_ent [ 17 others ] | 107063 (36.5%) 68427 (23.3%) 38744 (13.2%) 32311 (11.0%) 14584 (5.0%) 9744 (3.3%) 6048 (2.1%) 5307 (1.8%) 2747 (0.9%) 1600 (0.5%) 6669 (2.3%) | 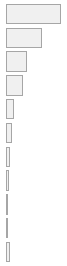 | 293244 (98.7%) | 3743 (1.3%) |
| 96 | Mother Occupational Code Level1 [factor] | 1. Military_occupations_and_ 2. OutsideWorkforce 3. Sales_and_service_occupat 4. College_professions 5. Office_jobs 6. Academic_professions 7. Cleaners 8. Leaders 9. Process_and_machine_opera 10. Handcrafted 11. Farmers_Fishermen | 64387 (26.0%) 49315 (19.9%) 47180 (19.1%) 37160 (15.0%) 13810 (5.6%) 13684 (5.5%) 8384 (3.4%) 6464 (2.6%) 4178 (1.7%) 2306 (0.9%) 785 (0.3%) | 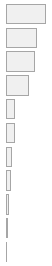 | 247653 (83.4%) | 49334 (16.6%) |
| 97 | Mother Occupational Code Level2 [factor] | 1. OutsideWorkforce 2. Professions_within_person 3. Sales_professions 4. Occupations_within_cultur 5. Office_workers 6. Healthrelated_professions 7. Employees_in_finance 8. Cleaners_etc 9. ICT_consultants 10. Administrative_and_mercan [ 20 others ] | 51740 (27.9%) 26121 (14.1%) 21059 (11.3%) 15718 (8.5%) 11254 (6.1%) 9497 (5.1%) 9059 (4.9%) 7727 (4.2%) 4987 (2.7%) 4675 (2.5%) 23764 (12.8%) | 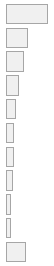 | 185601 (62.5%) | 111386 (37.5%) |
| 98 | Mother Occupational Code Level3 [factor] | 1. OutsideWorkforce 2. Shop_assistants 3. Waiters_and_bartenders 4. Professions_in_social_wor 5. Office_workers 6. Careers_in_alternative_me 7. Chefs 8. Agents_and_brokers 9. Occupations_within_financ 10. Frisrer [ 66 others ] | 53415 (33.1%) 21058 (13.1%) 17007 (10.5%) 8259 (5.1%) 7933 (4.9%) 6974 (4.3%) 4572 (2.8%) 4466 (2.8%) 4069 (2.5%) 3336 (2.1%) 30163 (18.7%) | 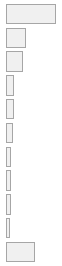 | 161252 (54.3%) | 135735 (45.7%) |
| 99 | Father Employment Status [factor] | 1. WageEarner 2. OutsideWorkforce 3. Independent 4. Free 5. Qualification | 251508 (85.3%) 21816 (7.4%) 16827 (5.7%) 4211 (1.4%) 615 (0.2%) | 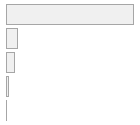 | 294977 (99.3%) | 2010 (0.7%) |
| 100 | Father Hours Worked Category [factor] | 1. 30plus 2. 19hrs 3. OutsideWorkforce 4. 20_29hrs | 253027 (87.4%) 21307 (7.4%) 7871 (2.7%) 7151 (2.5%) | 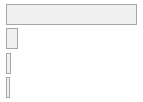 | 289356 (97.4%) | 7631 (2.6%) |
| 101 | Father Industry Sector1987 [factor] | 1. Private_companies_with_li 2. Personal_entrepreneur 3. The_State_and_Social_Secu 4. Municipalities 5. State-owned_enterprises 6. OutsideWorkforce 7. Private_consumer-oriented 8. Independent_municipal_ent 9. Personal_enterprises_etc 10. County_municipalities [ 17 others ] | 180535 (62.4%) 25205 (8.7%) 23496 (8.1%) 17953 (6.2%) 10823 (3.7%) 7995 (2.8%) 4773 (1.7%) 3970 (1.4%) 3436 (1.2%) 2864 (1.0%) 8038 (2.8%) |  | 289088 (97.3%) | 7899 (2.7%) |
| 102 | Father Occupational Code Level1 [factor] | 1. Military_occupations_and_ 2. Handcrafted 3. College_professions 4. Process_and_machine_opera 5. Sales_and_service_occupat 6. Academic_professions 7. OutsideWorkforce 8. Leaders 9. Office_jobs 10. Cleaners 11. Farmers_Fishermen | 43372 (17.4%) 39455 (15.9%) 36816 (14.8%) 26398 (10.6%) 22742 (9.1%) 21503 (8.6%) 20586 (8.3%) 15452 (6.2%) 10465 (4.2%) 10411 (4.2%) 1639 (0.7%) |  | 248839 (83.8%) | 48148 (16.2%) |
| 103 | Father Occupational Code Level2 [factor] | 1. OutsideWorkforce 2. Metal_and_machine_workers 3. Occupations_within_cultur 4. Construction_workers 5. Ingenirer_mv 6. Sales_professions 7. Realists 8. Administrative_and_mercan 9. Transport_workers_and_ope 10. Professions_within_person [ 20 others ] | 27204 (12.9%) 18434 (8.7%) 17575 (8.3%) 16391 (7.7%) 14523 (6.9%) 12618 (6.0%) 12083 (5.7%) 11755 (5.6%) 11484 (5.4%) 10123 (4.8%) 59359 (28.1%) |  | 211549 (71.2%) | 85438 (28.8%) |
| 104 | Father Occupational Code Level3 [factor] | 1. OutsideWorkforce 2. Shop_assistants 3. Professions_in_social_wor 4. Construction_workers 5. Ingenirer 6. Was 7. Mechanics_and_repairmen 8. Data_recorders_and_stenog 9. Biological_professions_et 10. Civil_engineers_except_el [ 64 others ] | 33784 (18.4%) 12618 (6.9%) 11380 (6.2%) 10742 (5.8%) 9712 (5.3%) 8366 (4.5%) 7590 (4.1%) 6441 (3.5%) 5877 (3.2%) 5712 (3.1%) 71790 (39.0%) |  | 184012 (62.0%) | 112975 (38.0%) |
| 105 | Mother Residence Settlement Urbanicity [factor] | 1. T 2. S 3. U | 246585 (83.0%) 49565 (16.7%) 837 (0.3%) |  | 296987 (100.0%) | 0 (0.0%) |
| 106 | Father Residence Settlement Urbanicity [factor] | 1. T 2. S 3. U | 244123 (82.2%) 51858 (17.5%) 1006 (0.3%) |  | 296987 (100.0%) | 0 (0.0%) |
| 107 | Mother Work Location Municipality [factor] | 1. OutsideWorkforce 2. 4601 3. 5001 4. 3024 5. 4204 6. 3005 7. 5401 8. 1507 9. 1108 10. 3004 [ 271 others ] | 44784 (21.1%) 19893 (9.4%) 11588 (5.5%) 7245 (3.4%) 5059 (2.4%) 4153 (2.0%) 4007 (1.9%) 3763 (1.8%) 3756 (1.8%) 3486 (1.6%) 104541 (49.2%) |  | 212275 (71.5%) | 84712 (28.5%) |
| 108 | Father Work Location Municipality [factor] | 1. OutsideWorkforce 2. 4601 3. 5001 4. 3024 5. 4204 6. 1108 7. 3005 8. 5401 9. 1507 10. 3025 [ 271 others ] | 25493 (12.3%) 21652 (10.4%) 12960 (6.3%) 9361 (4.5%) 5786 (2.8%) 4203 (2.0%) 4199 (2.0%) 4152 (2.0%) 3839 (1.9%) 3730 (1.8%) 111838 (54.0%) |  | 207213 (69.8%) | 89774 (30.2%) |
| 109 | Mother Residence Municipality [factor] | 1. 0301 2. 1201 3. 1601 4. 1103 5. 0219 6. 1001 7. 1102 8. 1902 9. 0106 10. 0602 [ 434 others ] | 51628 (17.5%) 21171 (7.2%) 11986 (4.1%) 9322 (3.2%) 7011 (2.4%) 4575 (1.5%) 4510 (1.5%) 4304 (1.5%) 3745 (1.3%) 3596 (1.2%) 173888 (58.8%) |  | 295736 (99.6%) | 1251 (0.4%) |
| 110 | Father Residence Municipality [factor] | 1. 0301 2. 1201 3. 1601 4. 1103 5. 0219 6. 1001 7. 1102 8. 1902 9. 0106 10. 0602 [ 429 others ] | 50603 (17.4%) 20928 (7.2%) 11803 (4.1%) 9240 (3.2%) 6860 (2.4%) 4536 (1.6%) 4409 (1.5%) 4143 (1.4%) 3725 (1.3%) 3608 (1.2%) 170988 (58.8%) |  | 290843 (97.9%) | 6144 (2.1%) |

### SSB dropped categories

For the prediction of baseline and follow-up participation, categories with fewer than 5,939 individuals were dropped. For the prediction of continued participation, categories with fewer than 1,723 individuals were dropped *(See supplementary file 4; Table S3)*.

###

### Norwegian Education Registry data: National test scores

####

#### Figure S2. Distributions of national test scores across Mathematics and English.

###

###

### Norwegian Control and Payment of Health Reimbursements Database (KUHR): Diagnostic codes

We also estimated the prevalences of and associations with probable anxiety and depressive disorders (including phobias) and probable ADHD. We describe these outcomes as probable because they are derived from primary care data and may be indicative of referrals for secondary care rather than diagnosis. Specifically, ICD10 and ICPC2 codes were recorded from the Norwegian Control and Payment of Health Reimbursements Database (KUHR). The KUHR includes diagnoses made across general practice (Fastlege), in outpatient clinics (Poliklinikk) and at the emergency department (Legevakt) between 2006 and 2023. Probable diagnoses of internalising (any anxiety [P74,F41.0, F41.1, F41.3, F41.8, F41.9], phobia [P79, F40.0, F40.1, F40.2, F40.8, F40.9, F42.0, F42.1, F42.2, F42.8, F42.9] or depression [P76, F32.0, F32.1, F32.2, F32.3, F32.8, F32.9, F33.0, F33.1, F33.2, F33.3, F33.4,F33.8, F33.9, F34.1, F34.8, F34.9, F38.0, F38.1, F38.8, F39, F41.2, F53.0]) and ADHD (any hyperkinetic diagnosis [P81, F90.0, F90.1, F90.8, F90.9]) were computed in the full eligible-to-participate population across the full range of dates (2006-2023).

# References

1. [Haghish EF. mlim: Multiple Imputation with Automated Machine Learning. 2022.](http://paperpile.com/b/ayHOwg/r3F6)

2. [Mayer M. Fast Imputation of Missing Values [R package missRanger version 2.6.1] [Internet]. *Comprehensive R Archive Network (CRAN)* 2024 [cited 2025 Nov 8]. Available from:](http://paperpile.com/b/ayHOwg/qH2N) [https://CRAN.R-project.org/package=missRanger](https://cran.r-project.org/package=missRanger)

3. [Stekhoven DJ, Bühlmann P. MissForest--non-parametric missing value imputation for mixed-type data. *Bioinformatics*. Oxford University Press (OUP); 2012 Jan 1;**28**(1):112–118.](http://paperpile.com/b/ayHOwg/JC18)

4. [Greenland S, Finkle WD. A critical look at methods for handling missing covariates in epidemiologic regression analyses. *Am J Epidemiol*. Oxford University Press (OUP); 1995 Dec 15;**142**(12):1255–1264.](http://paperpile.com/b/ayHOwg/jTvE)

5. [Little RJA, Rubin DB. Statistical analysis with missing data [Internet]. 3rd ed. Nashville, TN: John Wiley & Sons; 2025 [cited 2025 Nov 8]. Available from:](http://paperpile.com/b/ayHOwg/NGEM) <http://dx.doi.org/10.1002/9781119482260>

6. [Buuren S van. Flexible imputation of missing data, second edition [Internet]. 2nd ed. London, England: Taylor & Francis; 2021 [cited 2025 Nov 8]. Available from:](http://paperpile.com/b/ayHOwg/ZVIJ) <http://dx.doi.org/10.1201/9780429492259>

7. [Rubin DB. Multiple Imputation for Nonresponse in Surveys. 99th ed. Rubin DB, editor. Nashville, TN: John Wiley & Sons; 1987.](http://paperpile.com/b/ayHOwg/0aMZ)
