# Supplementary material for: Quantifying and adjusting for selection biases in the Norwegian Mother, Father and Child Cohort Study using population-wide individual-level registry information
Source: Int J Epidemiol. 2026 Jul 25;55(4):dyag122. doi: 10.1093/ije/dyag122 (PMC13401474; doi:10.1093/ije/dyag122)
Supplement: dyag122_Supplementary_Data [file dyag122_supplementary_data.zip › ije-2025-04-0715-File010.docx]

Quantifying and adjusting for selection biases in the Norwegian Mother, Father and Child Cohort Study using population-wide individual-level registry information

Supplementary file 3

[Statistical modeling 2](#_Toc233534255)

[Single imputation 2](#_Toc233534256)

[Identifying predictors of participation 3](#_Toc233534257)

**[Figure S7. Prediction of initial participation from the population](#_Toc233534258)** [5](#_Toc233534258)

[**Figure S8. Prediction of continued participation from the population** 6](#_Toc233534259)

[**Figure S9. Prediction of continued participation from baseline** 7](#_Toc233534260)

[Unweighted and weighted estimation of sample characteristics 8](#_Toc233534261)

[Unweighted and weighted estimation of outcome-exposure relationships 9](#_Toc233534262)

[**Figure S10.** Pairwise Pearson correlations between the three sets of predicted probabilities and inverse probability weights 10](#_Toc233534263)

[Quantifying biases and comparing adjustment strategies in MoBa 10](#_Toc233534264)

## Statistical modeling

### Single imputation

Where we had incomplete data on predictors of participation (S.Table 2), we performed single imputation of missing values using random forest regression and classification models using [mlim](https://cran.r-project.org/web/packages/mlim/mlim.pdf) [^1^](https://paperpile.com/c/ayHOwg/r3F6). We used the mlim::mlim.preimpute function with its default settings, which implements a multivariate iterative imputation procedure based on Random Forest, leveraging the high-performance missRanger package [^2^](https://paperpile.com/c/ayHOwg/qH2N), based on the missForest algorithm [^3^](https://paperpile.com/c/ayHOwg/JC18). First, all missing values in the dataset are filled with a preliminary estimate (e.g., mean for continuous, mode for categorical). The algorithm then iteratively models each variable containing missing data as a dependent variable, using all other variables in the dataset as predictors. For each variable, a Random Forest model is trained on the subset of observed values and is then used to predict its missing values. These predictions replace the initially imputed values. This cycle is repeated for all variables over several iterations until the imputed values stabilise (i.e., the difference between imputation steps falls below a convergence threshold), resulting in a single completed dataset for analysis. We opted for single imputation of missing values, because of the large sizes of the prediction datasets (N=296,987; predictors≈220), and complexities associated with pooling estimates across the downstream analyses. Single imputation allows us to estimate one prediction model per participation outcome, to derive a single weight, which can be applied once in each downstream analysis to demonstrate the impact of selection bias on the MoBa sample, to highlight the issue of selection bias to researchers using this dataset.

While single imputation provides a complete dataset for analysis and avoids the biases associated with listwise deletion, it has notable limitations. Simple methods for handling missing covariates can be highly sensitive to model misspecification and may lead to biased parameter estimates and invalid conclusions [^4^](https://paperpile.com/c/ayHOwg/jTvE). Single imputation replaces each missing value with only one estimate, failing to account for the statistical uncertainty in the imputation process [^5^](https://paperpile.com/c/ayHOwg/NGEM). As such, subsequent analyses may underestimate the standard errors of parameter estimates, leading to overly narrow confidence intervals and potentially inflated Type I error rates [^6^](https://paperpile.com/c/ayHOwg/ZVIJ). Furthermore, while Random Forest is a flexible non-parametric method, its performance degrades if the missing data mechanism is Missing Not At Random (MNAR) and not accounted for in the model [^3^](https://paperpile.com/c/ayHOwg/JC18). For a more robust approach that includes imputation uncertainty into final estimates, Multiple Imputation (MI) is recommended. By generating m>1 imputed datasets and pooling the final estimates (means, outcome exposure associations using Rubin's rules [^7^](https://paperpile.com/c/ayHOwg/0aMZ), standard errors and confidence intervals better reflect the uncertainty due to missing data. Despite these limitations, single imputation was deemed appropriate for the current study because our primary objective is descriptive—specifically, to assess the impact of representativeness and weighting—rather than to estimate causal effects where precise standard errors for hypothesis testing are critical.

### Identifying predictors of participation

Logistic regression models the probability of an outcome using the logistic function:

$P_{y=1} = \frac{1}{1+e^{-(z)}}$, where z is the linear combination of predictors, *z* = *β*₀ + *β*₁*x*₁ + ... + *β*ₙ*x*ₙ

The aim is to compute the coefficients (*β*) that maximise the likelihood of observing the data. The likelihood function represents the overall probability of observing the data given the model, and is the product of the predicted probabilities for all observations. The coefficients that maximize the log-likelihood (LL) are the best fitting parameters for the model.

LL = Σ*ᵢ* log ( *P* ( *yᵢ* | *xᵢ*, *β* ))

Penalized regression models use a penalty function to shrink the predictor coefficients. The Ridge [(Hoerl and Kennard 1970)](https://paperpile.com/c/UUWht4/9i2yI) penalty (λ₁) shrinks coefficients towards zero, which helps with multicollinearity and improves the stability of the model. Here, the loss function is defined as:

-LL + λ Σ *βᵢ*², where λ is the tuning parameter and λ Σ *βᵢ*² is the L1 shrinkage penalty.

The least absolute shrinkage and selection operator (LASSO; [(Tibshirani 1996)](https://paperpile.com/c/UUWht4/D3pi9)) penalty (λ₂) performs feature selection by forcing some coefficients to become exactly zero, which leads to a more sparse model with fewer predictors. Here the loss function is defined as:

-LL + λ Σ | *βᵢ* |, where λ is the tuning parameter and λ Σ | *βᵢ* | is the L2 shrinkage penalty.

The elastic-net [(Zou and Hastie 2005)](https://paperpile.com/c/UUWht4/iJD5H) mixing parameter (𝛼) controls the relative contribution from the L1 and L2 penalty when determining the coefficients and respective loss function.

-LL + λ Σ ( 𝛼*βᵢ*² + (1-𝛼 | *βᵢ* |), where 𝛼=0 is equal to the L1 penalty and 𝛼=1 is equal to the L2 penalty and 𝛼 values in between (e.g. .001, .01, and .1) are mixed elastic-net penalty values.

In all models the tuning parameter (λ) is determined via cross-validation. Here the data is split into *k* folds (e.g. 10), where each fold is an inner validation set, and the remaining *k*-1 folds are used to train the model. The coefficients from the model are used to predict outcomes in the inner validation set. The vector of coefficients which maximises the LL is selected as the optimal model. This is performed iteratively with varying λ values. For each λ value, the overall performance is computed as the average loss across the k-folds.

In practice, a matrix of predictors was restructured using R stats::model.matrix.lm and saved as file-backed matrices using bigstatsr::as_FBM. An elastic-net regression model (‘bigstatsr::big_spLogReg’) was then used to predict each of the participation outcomes (initial, continued). We used cross-model selection and averaging to automatically perform a procedure similar to cross-validation to choose hyper-parameters 𝜆 and 𝛼 of the elastic net regularization. This was performed in a training sample using 10-fold-leave-one-out iterations across five 𝛼 values (1, .1, .01, .001, .0001). The best fitting model was the one with the combination of 𝛼 and 𝜆 values which minimises the loss function. Each model was evaluated from visual inspection of plots (loss per validation set, classification accuracy, predictor importance) and indicators of prediction accuracy (AUC) in a held-out test sample (10% of total sample).

| **Figure S7. Prediction of initial participation from the population** **A.** Loss for each validation set across the five models (𝛼 values: 1, .1, .01, .001, .0001); **B.** Predicted probabilities of participation computed from the best performing model; **C.** Top 20 predictors from the best performing model, ranked by importance. There were 224 predictors in the full model |
| --- |
| 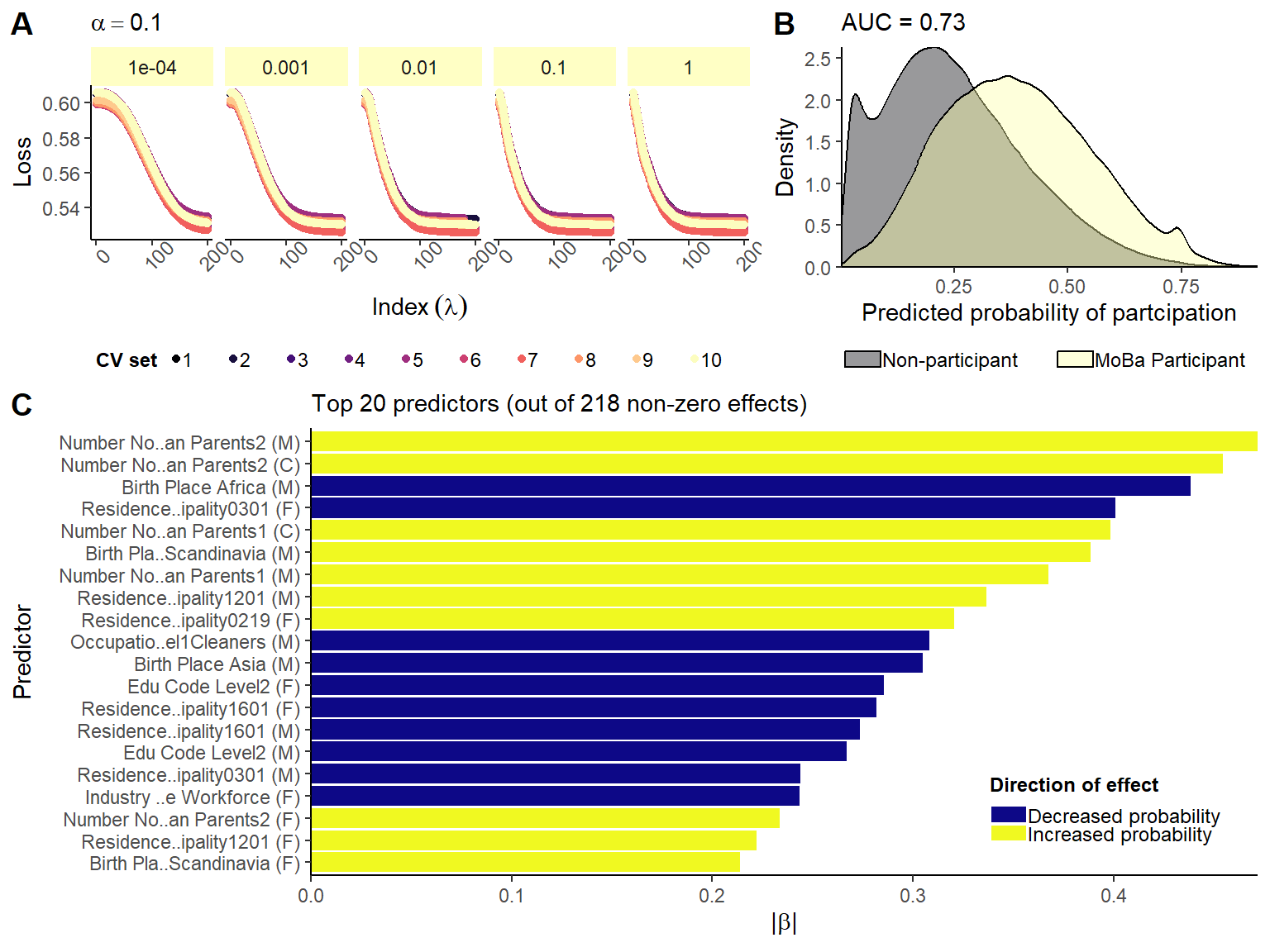 |
| ***[A] Facets (alpha)*** *display models with different elastic-net mixing parameters, which control the relative contribution from the LASSO (L1) and the Ridge (L2) penalty. An alpha of 1 is the LASSO penalty (forces some coefficients to be exactly zero), an alpha of 0 (or close to 0 i.e. 1e-4) is Ridge regression, and values in between are a mix called Elastic Net (.001, .01, and .1).* ***X-axis (Index):*** *represents different values of the* ***lambda*** *tuning parameter. Lambda controls the strength of the penalty in the penalized regression. Higher lambda means a stronger penalty (more coefficients shrunk towards zero).* ***Y-axis (Loss):*** *measures how well the model predicts the outcome on the validation set (the fold that was held out during training). Lower loss is better.* ***Coloured lines*** *correspond to the cross-validation subset (K=10). The lambda value (x-axis) where the average loss (across the 10 colored lines) is minimized represents the optimal penalty strength for the corresponding alpha.* |

#### **Figure S8. Prediction of continued participation from the population**

**A.** Loss for each validation set across the five models (𝛼 values: 1, .1, .01, .001, .0001); **B.** Predicted probabilities of participation computed from the best performing model; **C.** Top 20 predictors from the best performing model, ranked by importance. There were 225 predictors in the full model

| 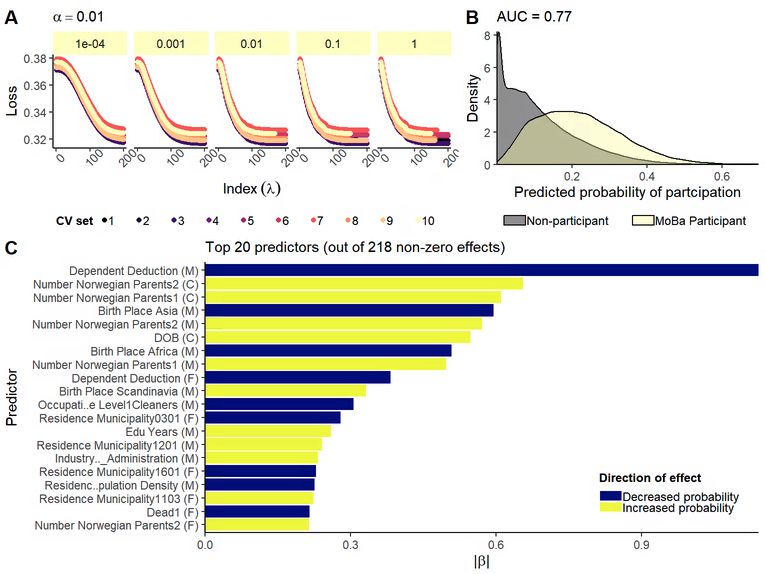 |
| --- |
| **[A] Facets (alpha)** display models with different elastic-net mixing parameters, which control the relative contribution from the LASSO (L1) and the Ridge (L2) penalty. An alpha of 1 is the LASSO penalty (forces some coefficients to be exactly zero), an alpha of 0 (or close to 0 i.e. 1e-4) is Ridge regression, and values in between are a mix called Elastic Net (.001, .01, and .1). **X-axis (Index):** represents different values of the **lambda** parameter. Lambda controls the strength of the penalty in your penalized regression. Higher lambda means a stronger penalty (more coefficients shrunk towards zero). **Y-axis (Loss):** measures how well the model predicts the outcome on the validation set (the fold that was held out during training). Lower loss is better. Common loss functions for logistic regression include deviance or misclassification error. **Coloured lines** correspond to the cross-validation subset (K=10). The lambda value (x-axis) where the average loss (across the 10 colored lines) is minimized represents the optimal penalty strength for the corresponding alpha. |

#### **Figure S9. Prediction of continued participation from baseline**

**A.** Loss for each validation set across the five models (𝛼 values: 1, .1, .01, .001, .0001); **B.** Predicted probabilities of participation computed from the best performing model; **C.** Top 20 predictors from the best performing model, ranked by importance. There were 225 predictors in the full model

| 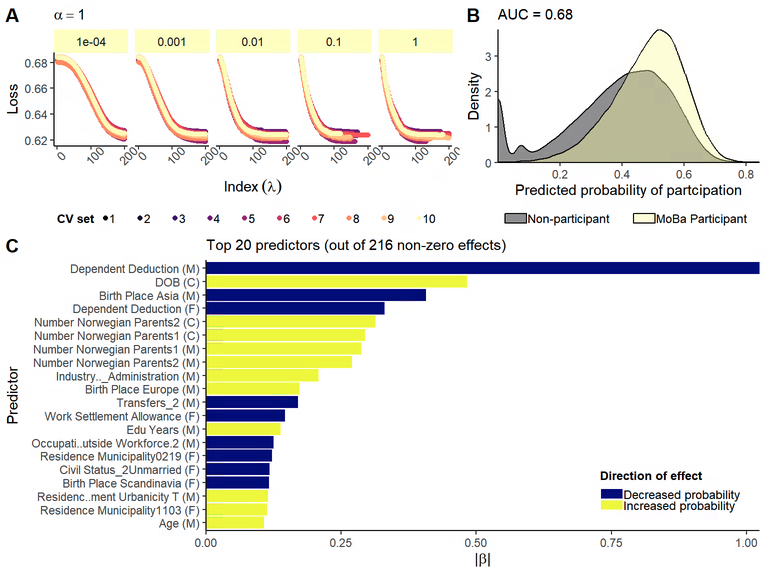 |
| --- |
| ***[A] Facets (alpha)*** *display models with different elastic-net mixing parameters, which control the relative contribution from the LASSO (L1) and the Ridge (L2) penalty. An alpha of 1 is the LASSO penalty (forces some coefficients to be exactly zero), an alpha of 0 (or close to 0 i.e. 1e-4) is Ridge regression, and values in between are a mix called Elastic Net (.001, .01, and .1).* ***X-axis (Index):*** *represents different values of the* ***lambda*** *parameter. Lambda controls the strength of the penalty in your penalized regression. Higher lambda means a stronger penalty (more coefficients shrunk towards zero).* ***Y-axis (Loss):*** *measures how well the model predicts the outcome on the validation set (the fold that was held out during training). Lower loss is better. Common loss functions for logistic regression include deviance or misclassification error.* ***Coloured lines*** *correspond to the cross-validation subset (K=10). The lambda value (x-axis) where the average loss (across the 10 colored lines) is minimized represents the optimal penalty strength for the corresponding alpha.* |

### Unweighted and weighted estimation of sample characteristics

We computed means or proportions, and variances for sociodemographic variables from the population registry data, comparing: i. The population parameter; ii. Unweighted estimates from the sample; iii. Weighted estimates from the sample. Unweighted and weighted means or proportions ($x$,$x$_w_) and variances (*σ^2^,σ^2^_w_*) or standard errors (SE of proportions) were computed as:

$\underline{x}=\frac{\Sigma(x_{i})}{n}$, $\underline{x}_{w}=\frac{\Sigma(w_{i}x_{i})}{\Sigma(w_{i})}$,

${\underline{\sigma^{2}}}=\frac{\Sigma(x_{i}- \underline{x})^{2}}{n - 1}$, ${{\underline{\sigma^{2}}}_{w}}=\frac{\Sigma w_{i}(x_{i}- \underline{x})^{2}}{\Sigma(w_{i})-1}$,

$SE=\sqrt{\frac{p(1-p)}{n}}$, ${SE}_{w}=\sqrt{\frac{p_{w}(1-p_{w})}{n_{eff}}}$, where $n_{eff}=\frac{\Sigma(w_{i})^{2}}{\Sigma({w_{i}}^{2})}$

### Unweighted and weighted estimation of outcome-exposure relationships

We also used univariable Ordinary Least Squares (OLS) and Weighted Least Squares (WLS) regression to compare outcome-exposure effects between sociodemographic variables and health outcomes. OLS regression can be expressed as:

*y_i_ = β_0_ + β_1_x_i_ + ε_i_* , where *y_i_* is the dependent variable, *x_i_* is the independent variable, *β_0_* is the intercept, *β_1_* is the slope, and *ε_i_* is the error term for the i-th observation. Here we assume that all observations have equal variance and are equally important. The best model is that which minimizes the sum of the squared vertical distances between the observed and predicted values:
 *Σ(y_i_ - ŷ_i_)²* , where *ŷ_i_* is the predicted value of *y_i_* based on the estimated model, and the coefficients are computed as:

*β_1_ =* $\frac{\Sigma(x_{i}- \underline{x})(y_{i}- \underline{y})}{\Sigma(x_{i}- \underline{x})^{2}}$ and *β_0_ = ȳ - β_1_x̄* , where *x̄* is the mean of *x* and *ȳ* is the mean of *y.*

Weighted Least Squares (WLS) regression assigns different weights to each observation to assign greater or lesser importance to the observation. Here we are upweighting observations for individuals who share characteristics with individuals who are less likely to participate, to improve sample representativeness. The model is the same as for OLS, but instead we are aiming to minimise:
 *Σ w_i_(y_i_ - ŷ_i_)²* , where *w_i_* is the weight assigned to the i-th observation and the coefficients are computed as:

*β_1_ =* $\frac{\Sigma w_{i}(x_{i}- \underline{x}_{w})(y_{i}- \underline{y}_{w})}{\Sigma w_{i}(x_{i}- \underline{x}_{w})^{2}}$ and *β_0_ = ȳ_w_ - β_1_x̄_w_* , where *x̄_w_* and *ȳ_w_* are the weighted means.

For unweighted analyses, the stats::glm R function was used and for weighted analyses the svy::svyglm R function was used.

#### **Figure S10.** Pairwise Pearson correlations between the three sets of predicted probabilities and inverse probability weights


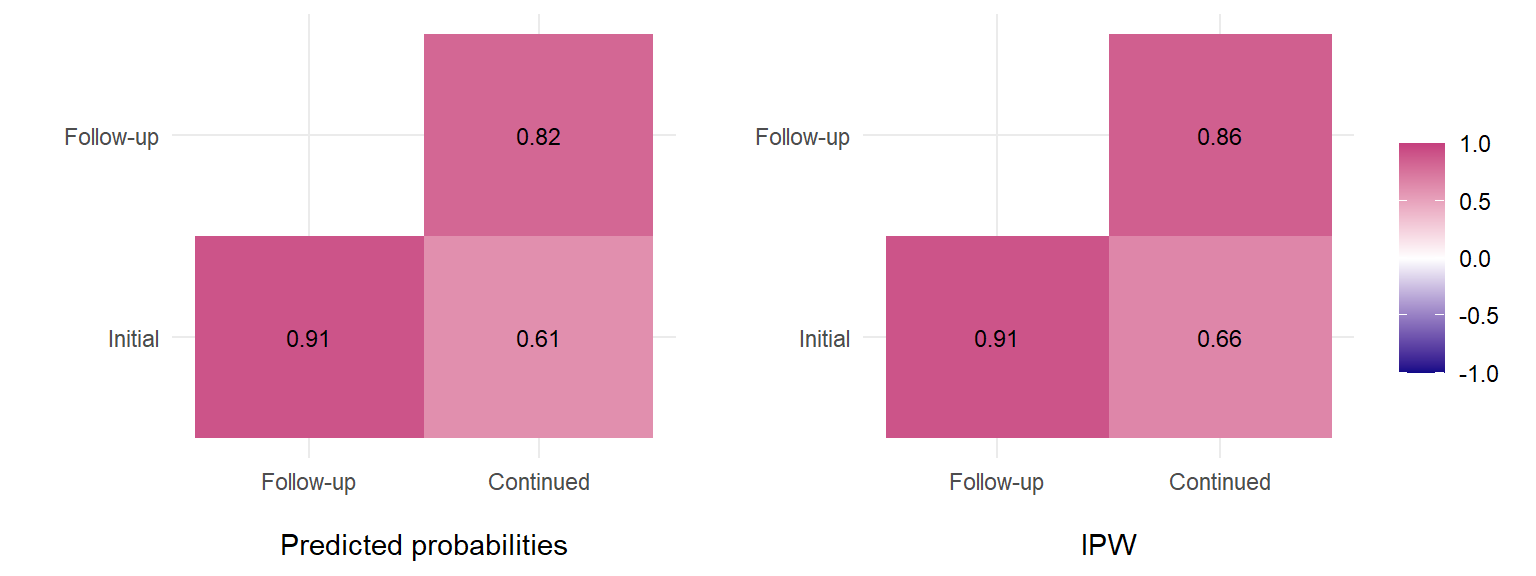


### Quantifying biases and comparing adjustment strategies in MoBa

Pairwise mean comparisons were tested using two-tailed two-sample z-tests, where, *x̄*, *σ* and *n* are the means, standard deviations and sample sizes of groups 1 & 2 respectively, and the degrees of freedom (df) were computed using the the Welch-Satterthwaite formula. Cohen’s *d* was computed as a standardised mean difference between the two values.

$Z = \frac{\underline{x}_{1} - \underline{x}_{2}}{\sqrt{\frac{\sigma_{1}^{2}}{n_{1}} + \frac{\sigma_{2}^{2}}{n_{2}}}}$ , with $df = \frac{\frac{\sigma_{1}^{2}}{n_{1}} + \frac{\sigma_{2}^{2}}{n_{2}}}{\frac{\left( \frac{\sigma_{1}^{2}}{n_{1}} \right)}{n_{1}-1}+ \frac{\left( \frac{\sigma_{2}^{2}}{n_{2}} \right)}{n_{2}-1}}$ , and $p=2 \times P( T\leq-|z|; df)$, $d = \frac{\underline{x}_{1} - \underline{x}_{2}}{\sqrt{\frac{{(n}_{1}-1)\sigma_{1}^{2} + {(n_{2}-1) \sigma_{2}^{2}}}{{(n}_{1} + {n_{2}-2)}}}}$

Pairwise variance comparisons were tested using F-tests:

$F = \frac{\sigma_{1}^{2}}{\sigma_{2}^{2}}$, with $p=P( F\leq F_{\alpha} ; {{df}_{1} =n}_{1}- 1, {{df}_{2} =n}_{2}- 1)$

Pairwise proportions were compared using the Wald statistic and χ^2^ tests with 1 df, and Cohen’s *h* was computed as a standardised difference between two proportions.

$W = \frac{(p_{1} - p_{2})^{2}}{{SE}_{1}^{2} + {SE}_{2}^{2}}$ *,* with $p=P(\chi^{2} \geq W ; df =1)$, $h = (2 arcsin\sqrt{p_{1}}) - (2 arcsin\sqrt{p_{2}})$

As such, bias in the unweighted & weighted sample estimates was quantified as the standardised difference between population and sample values. Standardised differences were quantified using Cohen’s d (means), Cohen’s h (proportions), F-statistic (variances) and Z-statistic (outcome-exposure effects) and statistical significance was assessed after correcting p-values for the number of effectively independent tests. Principal component analysis on the dataset with analysis variables indicated that 17 PCs were sufficient to explain 99.5% of the variance and thus our Bonferroni adjusted p-value was 0.05/17=0.00294. For each set of analyses (initial, continued), we computed the average standardised difference as a summary indicator of the bias.
